# Associations of Adiposity With Gut Microbiota Composition Among Adults—Results From a Federated Analysis of Individual Participant Data From Eight European Observational Studies

**DOI:** 10.1111/obr.70106

**Published:** 2026-03-01

**Authors:** Carolina Schwedhelm, Mariona Pinart, Sofia K. Forslund‐Startceva, Kolade Oluwagbemigun, Andreas Dötsch, Kristina Schlicht, Florian Schwarz, Sofia M. Siampani, Demetris Avraam, Maria De Angelis, Jildau Bouwman, Patrizia Brigidi, Giovanna Caderni, Francesco Maria Calabrese, Rafael R. C. Cuadrat, Carlotta De Filippo, Francesca De Filippis, Danilo Ercolini, Marco Fabbrini, Matthias Laudes, Ute Nöthlings, Serdar Özsezen, Itai Sharon, Matthias B. Schulze, Silvia Turroni, Francesco Vitali, Tobias Pischon, Katharina Nimptsch

**Affiliations:** ^1^ Max Delbrück Center for Molecular Medicine in the Helmholtz Association (MDC) Molecular Epidemiology Research Group Berlin Germany; ^2^ Experimental and Clinical Research Center, A Cooperation of Charité‐Universitätsmedizin Berlin and Max Delbrück Center for Molecular Medicine in the Helmholtz Association (MDC) Berlin Germany; ^3^ Charité‐Universitätsmedizin Berlin, Corporate Member of Freie Universität Berlin, Humboldt‐Universität zu Berlin Berlin Institute of Health Berlin Germany; ^4^ Host‐Microbiome Factors in Cardiovascular Disease Lab Max Delbrück Center for Molecular Medicine in the Helmholtz Association (MDC) Berlin Germany; ^5^ Structural and Computational Biology Unit European Molecular Biology Laboratory Heidelberg Germany; ^6^ German Centre for Cardiovascular Research (DZHK), Partner Site Berlin Berlin Germany; ^7^ Department of Nutrition and Food Sciences, Nutritional Epidemiology University of Bonn Bonn Germany; ^8^ Department of Physiology and Biochemistry of Nutrition Max Rubner‐Institut (MRI)—Federal Research Institute of Nutrition and Food Karlsruhe Germany; ^9^ Institute of Diabetes and Clinical Metabolic Research University of Kiel Kiel Germany; ^10^ Department of Molecular Epidemiology German Institute of Human Nutrition Potsdam‐Rehbruecke Nuthetal Germany; ^11^ Section of Epidemiology, Department of Public Health University of Copenhagen Copenhagen Denmark; ^12^ Department of Soil, Plant and Food Sciences University of Bari Aldo Moro Bari Italy; ^13^ Microbiology and Systems Biology Group Toegepast Natuurwetenschappelijk Onderzoek (TNO) Leiden the Netherlands; ^14^ Microbiomics Unit, Department of Medical and Surgical Sciences University of Bologna Bologna Italy; ^15^ NEUROFARBA Department, Pharmacology and Toxicology Section University of Florence Florence Italy; ^16^ Institute of Agricultural Biology and Biotechnology, National Research Council Pisa Italy; ^17^ Department of Agricultural Sciences University of Naples Federico II Portici Italy; ^18^ Task Force on Microbiome Studies University of Naples Federico II Naples Italy; ^19^ Unit of Microbiome Science and Biotechnology, Department of Pharmacy and Biotechnology University of Bologna Bologna Italy; ^20^ Migal‐Galilee Research Institute Kiryat Shmona Israel; ^21^ Faculty of Sciences and Technology Tel‐Hai Academic College Kiryat Shmona Israel; ^22^ Institute of Nutritional Science University of Potsdam Nuthetal Germany; ^23^ Biobank Technology Platform, Max‐Delbrueck‐Center for Molecular Medicine in the Helmholtz Association (MDC) Berlin Germany

**Keywords:** body mass index, federated analysis, fiber intake, gut microbiota, observational studies

## Abstract

Gut microbiota may contribute to the adiposity‐associated disease risk, but human studies reported inconsistent associations of adiposity with gut microbiota composition. We examined associations of body mass index (BMI) with alpha diversity and relative microbial abundance at the phylum and genus taxonomic levels (based on 16S rRNA amplicon sequencing or metagenomics) among 7415 adults from eight European observational studies in a joint federated analysis of harmonized data using DataSHIELD. Higher BMI (per 5 kg/m^2^) was associated with lower alpha diversity (*β*: −0.05; 95% CI: −0.07, −0.03) and, on the phylum level, positively associated with Proteobacteria, but neither with Firmicutes nor Bacteroidetes nor their ratio, where high between‐study heterogeneity was observed. On the genus level, BMI was inversely associated with the relative abundance of *Faecalibacterium* of the Firmicutes phylum (*β*: −0.11; 95% CI: −0.14, −0.07) but positively with the odds of detection of *Dorea*, *Streptococcus*, and *Clostridium* (all three Firmicutes) as well as *Collinsella* (Actinobacteria). This federated analysis of multiple studies found lower alpha diversity, alongside depleted *Faecalibacterium*, as well as higher odds of detection of *Dorea*, *Streptococcus*, *Clostridium*, and *Collinsella* with higher adiposity. By combining data from diverse study populations using harmonized data and statistical methods, our analysis partly overcomes sources of heterogeneity that may explain previously observed inconsistencies.

## 1 Introduction

Adiposity, reflected by body mass index (BMI), has increased worldwide [1], and because of the expected parallel rise in metabolic diseases and other obesity‐related morbidities, it is becoming a major public health concern. A growing body of evidence is sustaining the interaction between the gut microbiota and adiposity [2, 3], with possible therapeutic value [4]. Although findings have been inconsistent in human studies [5, 6], it has been hypothesized that the gut microbiota composition not only plays a role in obesity onset but also intervenes in the higher disease risk of comorbidities among people with obesity [7, 8].

Alterations in the gut microbiota composition with increasing BMI that have been described include lower bacterial alpha diversity and a higher *Firmicutes* to *Bacteroidetes* (*F*:*B*) ratio at the phylum level [2, 9]. Although some animal and human studies support these hypotheses [10, 11, 12, 13, 14], other studies have found no associations [11, 15, 16, 17] or reported contradictory associations [9, 12, 18, 19, 20]. Also at the genus taxonomic level, observations from different studies have often been inconsistent [9].

Dietary fiber intake, which may interact with the human gut microbiota [21, 22, 23] and has been inversely associated with BMI [24, 25], is a potentially confounding factor in the BMI–gut microbiota association.

Previous systematic reviews [2, 26] and a systematic review with a literature‐based meta‐analysis [9] attribute the inconsistent results partly to differences in microbiome measurement techniques, including methodologies for the quantification of microorganisms, and to the diverse and often insufficient control for confounders in the individual studies [9]. In addition, BMI comparison groups across studies were heterogeneous [9]. Joint analysis of multiple studies with harmonized individual‐level data [27] may help overcome these sources of heterogeneity with consistent variable definitions, statistical methods, and confounder adjustment. Federated analysis grants the opportunity to analyze individual‐level data from multiple studies jointly in a flexible way while keeping the data strictly secure, and no need for physical data transfer [28].

We here investigated associations of BMI with gut microbiota composition (alpha diversity and relative abundance at the phylum and genus taxonomic levels) in eight European observational studies with high‐throughput microbiome sequencing techniques participating in the Intestinal Microbiomics Knowledge Platform (INTIMIC‐KP) [29] using DataSHIELD, a statistical platform for federated, privacy‐preserving analysis of individual‐level data from multiple studies [28].

## 2 Methods

### 2.1 Study Population

The observational studies included in this study were identified in the INTIMIC‐KP of the Joint Programming Initiative—A Healthy Diet for a Healthy Life (JPI HDHL) collaboration [29, 30]. Out of 10 eligible (cross‐sectional data, high‐throughput microbiota data, participant age ≥ 18 years) studies, seven observational studies (five population‐based, two disease‐based) and the control arm of one intervention study (healthy volunteers) participated (Table 1), contributing data from 7420 participants. This study was performed in line with the principles of the Declaration of Helsinki. Approval was granted by the local Ethics Committee of the participating studies (for details, please refer to the Supporting Information: Methods). Written informed consent was obtained from all individual participants of the participating studies.

**TABLE 1:** Observational studies from the INTIMIC‐KP consortium included in the federated analysis.^a^

Study	Country	Study design	Population	Gut microbiota			BMI assessment	Dietary assessment
				Number of participants with microbiota data	Method^b^	Taxonomic database^c^		
Diet4MicroGut [31]	Italy	Cohort	Population‐based	143	16S (V1–V3)	Greengenes	Self‐reported BMI	Food records
DONALD study [32]	Germany	Cohort	Population‐based	79	16S (V3–V4)	SILVA	Weight and height measured	Food records
EPIC‐Potsdam substudy [33]	Germany	Cohort	Population‐based	3299	16S (V3–V4)	SILVA	Weight and height measured	M24h‐R SQ‐FFQ
ErNst [34]	Germany	Cross‐sectional	Population‐based	107	16S (V4)	SILVA	Weight and height measured	SQ‐FFQ
FoCus [35]	Germany	Cohort	Disease‐based (obesity) & population‐based (for controls)	1524	16S (V1–V2)	RDP	Weight and height self‐reported/measured in a subset	SQ‐FFQ
MeaTIc [36]	Italy	Intervention	Healthy volunteers	85	16S (V3–V4)	SILVA	Weight and height measured	Food records
MetaCardis [37]	Multinational (Germany, France, Denmark)	Cross‐sectional	Disease‐based (CMD^6^)	1982	Shotgun	^d^	Weight and height measured	M24h‐R (subset of participants); SQ‐FFQ
Italian Elderly Cohort (NU‐AGE) [38]	Italy	Cohort	Population‐based	201	16S (V3–V4)	SILVA	Weight and height measured	Food records

Abbreviations: CMD, cardiometabolic disease; INTIMIC‐KP, Knowledge Platform on Food, Diet, Intestinal Microbiomics and Human Health; M24h‐R, multiple 24‐h recalls; SQ‐FFQ, semiquantitative food frequency questionnaire.

^a^ The general population includes both random and convenience sampling designs.

^b^ 16S: 16S rRNA gene amplicon sequencing (amplified variable regions), shotgun: shotgun metagenomic sequencing.

^c^ Database used for taxonomic units classification.

^d^ Algorithms and databases used for quantification and classification of taxa from shotgun reads based on the mOTU approach are described elsewhere [39].

### 2.2 Data Assessments

BMI expressed in kg/m^2^ was derived from measurements in six out of eight studies (Table 1). Gut microbiota composition was obtained from high‐throughput sequencing of stool samples (16S rRNA gene sequencing in seven studies; shotgun metagenomics in one study). Relative abundance (in percent, range: 0%–100%) was derived at phylum and genus level, respectively, and Shannon–Wiener diversity index (*H′* = −*Σ* (*p*_*i*_ × ln (*p*_*i*_)), where *p*_*i*_ is the proportion of the *i*th sequence variant relative to the total number of sequence variants observed, from here on referred to as Shannon index), a measure for alpha diversity accounting for the number of species (richness) and their relative abundance (evenness) [40] was calculated in each study separately. Five phyla and 25 genera were identified as candidate bacteria to be investigated as outcome variables based on the results of our previous systematic review [9] (see Table S1). In 2022, the International Code of Nomenclature of Prokaryotes was updated, and according to the new code, *Firmicutes* is referred to as *Bacillota*, and the correct naming of the other phyla would be *Bacteroidota*, *Pseudomonadota* (formerly Proteobacteria), *Actinobacteriota*, and *Verrucomicrobiota* [41]. For the purpose of this investigation, we chose to maintain the old nomenclature, i.e., the one used when studies were carried out.

Study participants' characteristics including sex, age, smoking status, education level, prevalent diseases, current use of medication, past use of antibiotics, and physical activity were obtained from study‐specific questionnaires (Table S1). Dietary variables, when available, were obtained from a food‐frequency questionnaire (ErNst [34], FoCus [35]) or from food records (Diet4MicroGut [31], DONALD [42], MeaTIc [36], and NU‐AGE [43]).

The data harmonization and upload processes were comparable to a previous federated analysis of multiple studies [44]. Details on the local and centralized data harmonization processes are described in the Supporting Information: Methods and in Table S1. Study partners uploaded the harmonized datasets to their local servers together with the corresponding data dictionaries and gave the analysts permission to access the data via DataSHIELD.

### 2.3 Statistical Analysis

Overall, studies uploaded data for 7420 participants, of whom participants with a Shannon index equal to zero were excluded, leaving a total of 7415 participants for analyses. Centralized data harmonization, derivation of variables, and statistical analyses were performed in R software (version 4.3.1, R Foundation for Statistical Computing, Vienna, Austria) using the federated data analysis platform DataSHIELD (dsBaseClient package, version 6.2) [28]. Individual participant data (IPD) remained on the servers of the contributing study locations and were analyzed from a central analysis computer [44].

Participants' characteristics were summarized using frequency and percentage for categorical variables, mean and standard deviation for (visually) approximately normally distributed continuous variables, and median and interquartile range (IQR) for visually skewed continuous variables. Spearman correlation coefficients between BMI and fiber intake were calculated. Generalized linear models (GLMs) were used to examine the cross‐sectional associations of BMI (independent variable) with gut microbiota composition (dependent variables). We performed meta‐analysis using IPD in a study‐level meta‐analysis (SLMA), also referred to as the two‐stage IPD approach [45], where the regression analysis was performed separately for each study, and then, the study‐specific results were combined using conventional meta‐analysis [28, 44]. For comparison, we additionally used virtual IPD analysis in a one‐stage approach [45], which refers to a full‐likelihood‐based methodology generating results equivalent to analyzing pooled data [46].

We applied linear regression with BMI as the independent variable and Shannon index or relative abundance (in percent, range 0%–100%) of prevalent taxa (present in > 90% of samples). All models were adjusted for age and sex, and one‐stage IPD models also for the study source. To address skewed distributions, linear regression models with the relative abundance of taxa as the outcome were log‐transformed after adding a pseudocount of 0.01%, which also has the advantage of being robust against outliers. Shannon index, Firmicutes, and Bacteroidetes distributions were not skewed and not transformed for analysis. Because of heteroscedasticity detected in some models by using nondisclosive regression plot diagnostics [47], we show robust standard errors based on the HC1 method [48] with their corresponding confidence intervals and *p*‐values for all models. For the *Prevotella*:*Bacteroides* (*P*:*B*) ratio, a previously described bimodal distribution [49] was observed (see Figure S1), so this outcome was modeled as a dichotomous variable in logistic regression (high vs. low *P*:*B* ratio). In addition, we applied fractional regression [50] to the relative abundance data of all taxa. This semiparametric approach has been previously applied to gut microbiota relative abundance data [51], is robust toward heteroskedasticity, and is easily interpretable, since the regression coefficients can be interpreted as odds ratios (ORs) corresponding to the chance that a certain sequence is assigned to certain phyla or genera. This method also accommodates less prevalent taxa that contain a substantial amount of zero values (indicating that the corresponding taxa were not observed in the sample) (see Table S3). Taxa present in < 10% of the samples were excluded from all analyses.

Between‐study heterogeneity was assessed using the Chi^2^‐test and *I* ^2^ statistics (≥ 50% referring to substantial heterogeneity) [52]. Using the “leave‐one‐study‐out” approach, we evaluated the impact on summary results and between‐study heterogeneity [53]. We estimated the association of a 5‐unit higher BMI (an increment frequently used in epidemiology to indicate health‐relevant BMI differences) with gut microbiota composition (Shannon index and the relative abundance of candidate taxa on the phylum and genus level). Moreover, we investigated the role of fiber intake by additionally adjusting for it. We corrected for multiple testing according to the Benjamini–Hochberg method (false discovery rate, FDR) [54] with an FDR of 0.05 and accounting for 36 tests (12 linear regressions, 1 logistic regression, and 23 fractional regressions).

## 3 Results

### 3.1 Sample Characteristics

Participant characteristics are summarized in Table 2. Diet4MicroGut had the study population with the lowest BMI, including 87% of the participants with BMI < 25 and 0% with BMI > 30, and MetaCardis had (by design) the study population with the highest obesity prevalence (52%). Median fiber intake varied between 14.8 g/day in DONALD and 37.7 g/day in Diet4Microgut. BMI was weakly inversely correlated with energy‐adjusted fiber residuals in all studies except DONALD (Table S2).

**TABLE 2:** Baseline characteristics of the participants from eight European studies included in the federated analysis (*N* = 7415).^a^

Characteristics	Diet4MicroGut *N* = 143	DONALD *N* = 79	EPIC‐Potsdam substudy *N* = 3299	ErNst *N* = 107	FoCus *N* = 1520^b^	MeaTIc *N* = 85	MetaCardis *N* = 1982	NU‐AGE *N* = 200^b^
Sociodemographic								
Sex: Female	83 (58.0)	51 (64.6)	1907 (57.8)	53 (49.5)	957 (63.0)	60 (70.6)	981 (49.5)	100 (50)
Age, year	38.03 ± 9.44	25.03 ± 6.09	68.39 ± 8.11	49.15 ± 17.36	51.71 ± 14.21	37.15 ± 10.48	56.54 ± 12.24	71.20 ± 3.82
BMI, kg/m^2^	21.93 ± 2.29	24.12 ± 4.85	27.18 ± 4.78	24.51 ± 4.11	30.87 ± 9.80	24.62 ± 4.21	32.53 ± 9.06	27.05 ± 3.78
< 25	125 (87.4)	57 (72.2)	1164 (35.3)	65 (60.8)	507 (33.4)	50 (58.8)	430 (21.7)	61 (30.5)
25 to < 30	18 (12.6)	14 (17.7)	1374 (41.6)	33 (30.8)	418 (27.5)	26 (30.6)	504 (25.4)	102 (51.0)
≥ 30	0 (0)	8 (10.1)	761 (23.1)	9 (8.4)	595 (39.1)	9 (10.6)	1025 (51.7)	37 (18.5)
Smoking status								NA
Current	19 (13.3)	12 (15.2)	300 (9.1)	7 (6.5)	274 (18.0)	0 (0)	275 (13.9)	—
Former	NA	11 (13.9)	1310 (39.7)	28 (26.2)	677 (44.5)	0 (0)	873 (44.0)	—
Never	NA	49 (62.0)	1655 (50.2)	72 (67.3)	521 (34.3)	85 (100)	808 (40.8)	—
Education, ≥ 12 years	NA	41 (51.9)	1495 (45.3)	NA	624 (41.1)	NA	812 (41.0)	NA
Prevalent cardiometabolic diseases^c^	0 (0)	NA	3266 (99.0)	18 (16.8)	667 (43.9)	54 (63.5)	1859 (93.8)	NA
Current use of medication^d^	0 (0)	NA	1354 (41.0)	7 (6.5)	331 (21.8)	3 (3.5)	1021 (51.5)	NA
Regular use of probiotics	0 (0)	NA	NA	NA	NA	0 (0)	NA	NA
Recent use of antibiotics^e^	143 (100)	19 (24.1)	10 (0.3)	8 (7.5)	NA	18 (21.2)	822 (41.5)	NA
Dietary intake								
Total energy, kcal/day	2359 [2140, 2618]	1555 [1401, 1703]	NA	1834 [1496, 2774]	2069 [1693, 2547]	1671 [1600, 1836]	NA	1735 [1477, 2010]
Carbohydrate intake, g/day	306.78 [271.17, 357.01]	193.93 [171.24, 222.44]	NA	249.88 [173.70, 354.31]	213.66 [170.44, 271.99]	206.5 [179.82, 232.27]	NA	226.50 [176.12, 259.27]
Fiber intake, g/day	37.65 [26.67, 47.47]	14.81 [12.91, 16.31]	NA	23.75 [16.39, 34.79]	20.83 [16.64, 26.02]	15.8 [13.30, 21.25]	NA	21.02 [15.38, 26.80]
Protein intake, g/day	75.21 [65.53, 90.00]	50.40 [44.76, 57.78]	NA	75.23 [56.15, 99.08]	75.31 [61.44, 94.31]	68.27 [62.35, 78.42]	NA	67.20 [57.71, 77.23]
Total fat intake, g/day	94.36 [82.00, 107.35]	58.80 [54.70, 67.70]	NA	66.11 [49.71, 87.25]	92.14 [74.78, 114.39]	71.5 [66.15, 77.50]	NA	59.62 [51.59, 70.88]
Saturated fatty acid intake, g/day	25.51 [16.68, 36.21]	27.29 [24.17, 31.14]	NA	29.92 [22.19, 38.37]	37.26 [29.38, 46.9]	18.3 [14.63, 20.91]	NA	18.64 [15.57, 23.50]
Monounsaturated fatty acid intake, g/day	51.20 [44.36, 58.08]	19.70 [17.52, 22.77]	NA	21.13 [16.01, 28.60]	15.61 [12.63, 19.56]	32.37 [27.92, 37.18]	NA	27.41 [22.23, 32.46]
Polyunsaturated fatty acid intake, g/day	15.57 [12.81, 19.81]	8.01 [6.93, 9.21]	NA	9.03 [6.48, 12.50]	32.34 [26.03, 40.51]	7.97 [6.67, 9.57]	NA	7.66 [6.28, 10.36]
Nondrinkers	28 (19.6)	0 (0)	NA	0 (0)	0 (0)	NA	NA	42 (21.0)
Alcohol, g/day	4.28 [0.89, 11.69]	0.55 [0.35, 1.69]	NA	5.47 [1.89, 9.52]	5.58 [1.93, 13.46]	NA	NA	5.40 [0.50, 13.98]
Gut microbiota composition								
Shannon–Wiener alpha diversity index	5.76 (0.95)	6.03 (0.53)	4.22 (0.36)	4.19 (0.38)	4.09 (0.56)	4.21 (0.38)	3.43 (0.58)	1.89 (0.40)
*F*:*B* ratio	1.60 [0.91, 2.96]	1.78 [1.43, 2.18]	1.40 [1.09, 1.82]	4.49 [2.71, 7.07]	1.02 [0.72, 1.50]	1.87 [1.23, 2.85]	0.50 [0.29, 0.84]	15.94 [6.52, 67.08]
*P*:*B* ratio, high (> 0.01)	75 (52.4)	0 (0)	94 (2.8)	48 (44.9)	653 (43.0)	5 (5.9)	837 (42.2)	107 (53.5)

Abbreviation: NA, not available.

^a^ Values are mean ± SD or median [25th, 75th percentiles] or *N* (%).

^b^ Participants with alpha diversity of zero were excluded from the analytical sample; *N* = 4 in FoCus and *N* = 1 in NU‐AGE.

^c^ Hypertension, diabetes, cardiovascular diseases, and dyslipidemia.

^d^ Statins, nonsteroidal anti‐inflammatory drugs, and/or diabetes medications. No data on NSAID available for MeaTIc and MetaCardis.

^e^ Study‐specific definitions: Diet4MicroGut: past 12+ months; ErNst: past 3, 6, or 12 months; DONALD and MeaTIc, past 6 months; EPIC‐Potsdam substudy, past 3 months; MetaCardis, past 5 years up to 3 months before enrollment.

The mean Shannon index varied between 3.43 (MetaCardis) and 4.22 (ErNst) in the majority of studies (EPIC‐Potsdam, ErNst, FoCus, MetaCardis, and MeaTIc), with three studies having substantially lower (NU‐AGE, 1.89) or higher (Diet4MicroGut, 5.76; DONALD, 6.03) alpha diversity. Table S3 shows characteristics by BMI categories.

Taxa of the assessed bacterial phyla were detected in at least 97% of all samples except for Verrucomicrobia (prevalence 66%) (Table S4). On the genus level, five of the 25 taxa were detected in > 90% of samples, and one taxon (*Rikenella*) was found in < 10% of samples and was excluded from further analyses.

### 3.2 Association of BMI With Alpha Diversity

**FIGURE 1:**
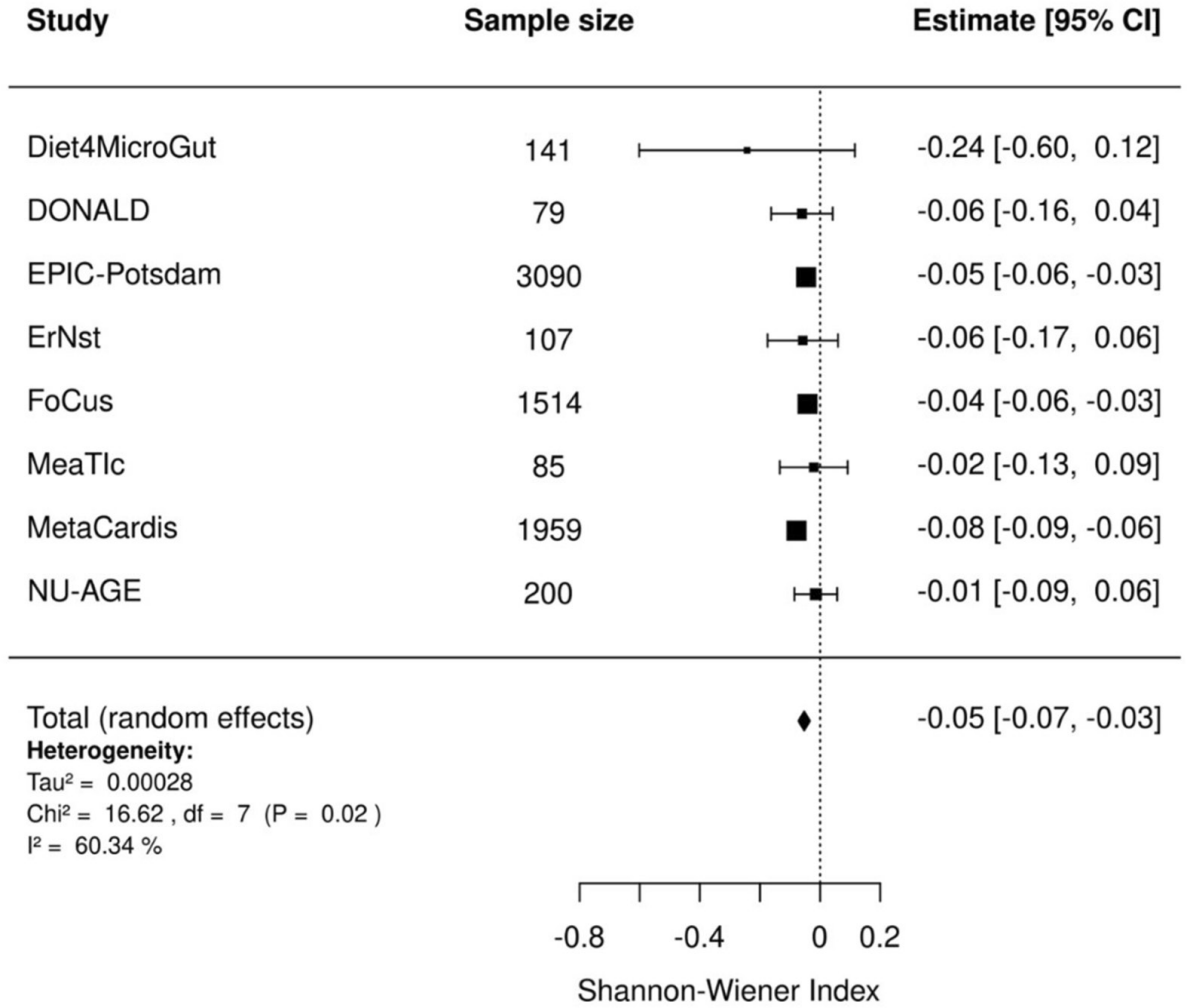
Forest plot of random‐effects study‐level meta‐analysis showing the mean difference in alpha diversity (Shannon‐Index) per 5‐unit BMI increment among adults from eight European studies, sex and age adjusted.

The age‐ and sex‐adjusted SLMA showed that a 5 kg/m^2^ higher BMI was associated with a lower Shannon index (*β*: −0.05; 95% CI: −0.07; −0.03, FDR‐adjusted *p* < 0.0001), with substantial between‐study heterogeneity (*I* ^2^ = 60%). Despite the heterogeneity, all effect estimates are consistent with an inverse association (Figure 1). Although excluding the EPIC‐Potsdam substudy from analysis reduced heterogeneity (*I* ^2^ = 0%), the results for the association between BMI and Shannon index were unchanged (data not shown).

### 3.3 Association of BMI With *F*:*B* and *P*:*B* Ratios

**FIGURE 2:**
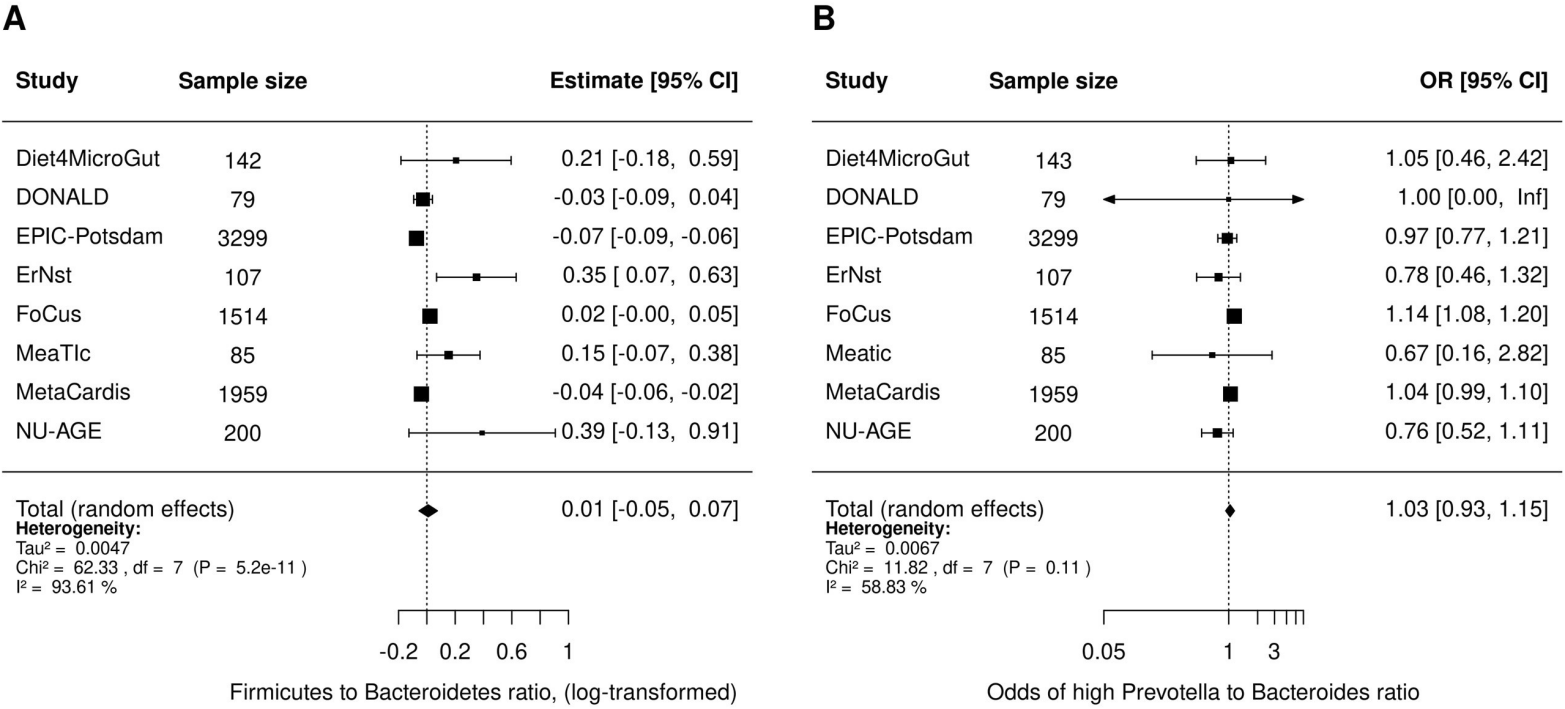
Forest plots of random‐effects study level meta‐analysis among adults from eight European studies, sex and age adjusted, showing (A) the mean difference in *Firmicutes* to *Bacteroidetes* ratio (log‐transformed) per 5‐unit BMI increment and (B) odds of high (> 0.01) *Prevotella* to *Bacter*.

Higher BMI was not associated with *F*:*B* ratio, with high heterogeneity (*I* ^2^ = 94%) and inconsistent results across studies (Figure 2A). Similarly, higher BMI was not associated with the odds of high versus low *P*:*B* ratio (Figure 2B).

### 3.4 Association of BMI With Relative Abundance of Prevalent Taxa

On the phylum level, higher BMI was not associated with the relative abundance of Firmicutes, Bacteroidetes, and Actinobacteria (Table 3), with inconsistent results and substantial heterogeneity (Figure S2). Excluding EPIC‐Potsdam substudy from the analysis resulted in lower between‐study heterogeneity for Firmicutes (*I* ^2^ = 1%) and a positive association with BMI (*β*: 0.40; 95% CI: 0.23, 0.58). Higher BMI was associated with higher relative abundance of the phylum Proteobacteria (*β*: 0.05; 95% CI: 0.01, 0.09), but not statistically significant after adjustment for multiple testing (FDR‐adjusted *p* = 0.07) (Table 3). On the genus level, higher BMI was not associated with the relative abundance of *Bacteroides*, *Parabacteroides* (both *Bacteroidetes* phylum), *Roseburia*, and *Ruminococcus* (both Firmicutes), and between‐study heterogeneity was substantial (*I* ^2^ > 50). A 5‐unit BMI increment was associated with lower relative abundance of *Faecalibacterium* (Firmicutes) (*β*: −0.11, 95% CI: −0.14, −0.07 on the log‐transformed scale, FDR‐adjusted *p* < 0.0001) with low heterogeneity (*I* ^2^ = 20%) (Table 3 and Figure S3).

**TABLE 3:** Random effects study level meta‐analyses of the associations between BMI (per 5‐unit increase) and alpha diversity, relative abundance of prevalent taxa at the phylum and genus level^a^,^b^ and *F*:*B* and *P*:*B* ratios.

Gut microbiome composition characteristic	Mean difference per 5 BMI (kg/m^2^)‐units increment^c^					
	*N*	*β* (95% CI)	Adjusted *p*‐value^d^	Heterogeneity *χ* ^2^	Heterogeneity *p*‐value	*I* ^2^ (%)
Alpha diversity	7175	−0.05 (−0.07; −0.03)	< 0.0001	16.62	0.02	60
*F*:*B* ratio^e^	7385	0.01 (−0.06; 0.08)	0.90	62.24	< 0.0001	94
More prevalent taxa (present in ≥ 90% of samples)						
Phylum						
*Actinobacteria* ^e^	7385	0.03 (−0.01; 0.06)	0.22	8.19	0.32	36
*Bacteroidetes*	7385	−0.76 (−2.34; 0.82)	0.55	117.91	< 0.0001	97
*Firmicutes*	7385	0.37 (−0.82; 1.57)	0.74	116.38	< 0.0001	96
*Proteobacteria* ^e^	7385	0.05 (0.01; 0.09)	0.07	12.33	0.09	53
Genus						
*Bacteroides* (Bacteroidetes)^e^	7386	−0.01 (−0.05; 0.04)	0.90	26.40	0.0004	69
*Parabacteroides* (Bacteroidetes)^e^	7386	−0.03 (−0.09; 0.03)	0.55	19.45	0.01	60
*Faecalibacterium* (Firmicutes)^e^	7386	−0.11 (−0.14; −0.07)	< 0.0001	12.62	0.08	20
*Roseburia* (Firmicutes)^e^	7386	0.03 (−0.02; 0.08)	0.37	14.17	0.05	53
*Ruminococcus* (Firmicutes)^e^	7386	−0.07 (−0.15; 0.01)	0.22	44.67	< 0.0001	78
				Odds per 5 BMI (kg/m^2^)‐units increment^f^		
	N	OR (95% CI)	Adjusted *p*‐value^c^	Heterogeneity *χ* ^2^	Heterogeneity *p*‐value	*I* ^2^ (%)
*P*:*B* ratio (high vs. low)^g^	7386	1.03 (0.93; 1.15)	0.74	11.82	0.11	58

Abbreviations: *F*:*B*, *Firmicutes* to *Bacteroidetes* ratio; *P*:*B*, *Prevotella* to *Bacteroides* ratio.

^a^ Participants with alpha diversity of zero were excluded; *N* = 4 in FoCus and *N* = 1 in NU‐AGE. Excluding taxa present in < 10% of samples.

^b^ In 2022, the International Code of Nomenclature of Prokaryotes was updated, and according to the new code, Firmicutes is referred to as Bacillota, and the correct naming of the other phyla would be Bacteroidota, Pseudomonadota (formerly Proteobacteria), Actinobacteriota, and Verrucomicrobiota [41]. We here chose to maintain the old nomenclature, i.e., the one used when studies were carried out.

^c^ Linear regression using generalized linear models with robust standard errors to address heteroscedasticity of residuals observed in some models. Dependent variables: Alpha diversity, relative abundance of prevalent taxa at the phylum/genus level; independent variables: BMI per 5 (kg/m^2^)‐unit increment plus sex and age (years) for adjustment.

^d^ Using Benjamini–Hochberg FDR controlling procedure.

^e^ Ratios and taxa were log‐transformed (natural logarithm). A pseudocount of 0.01% was added to relative abundance (all observations) to allow for log‐transformation in the presence of zeros.

^f^ Logistic regression using generalized linear models was fitted for estimating the probability of the binary outcome (high versus low *P*:*B* ratio or taxa as binary variable: taxa observed versus taxa not observed in sample). Dependent variable: *P*:*B* ratio; independent variables: BMI per 5 (kg/m^2^)‐unit increment plus sex and age (years) for adjustment.

^g^ *Prevotella* to *Bacteroides* (*P*:*B*) ratio was dichotomized, where values > 0.01 were categorized as high.

Results of the linear regression were mostly consistent between SLMA (two‐stage approach) and IPD (one‐stage approach), with some exceptions (Table S5): Associations were observed in the one‐stage approach but not in the two‐stage SLMA for *F*:*B* ratio, Bacteroidetes, and Actinobacteria; however, except for Actinobacteria, all these associations were characterized by high between‐study heterogeneity in the (two‐stage) SLMA analysis.

### 3.5 Results of the Fractional Regression for Both Prevalent and Less Prevalent Taxa

**FIGURE 3:**
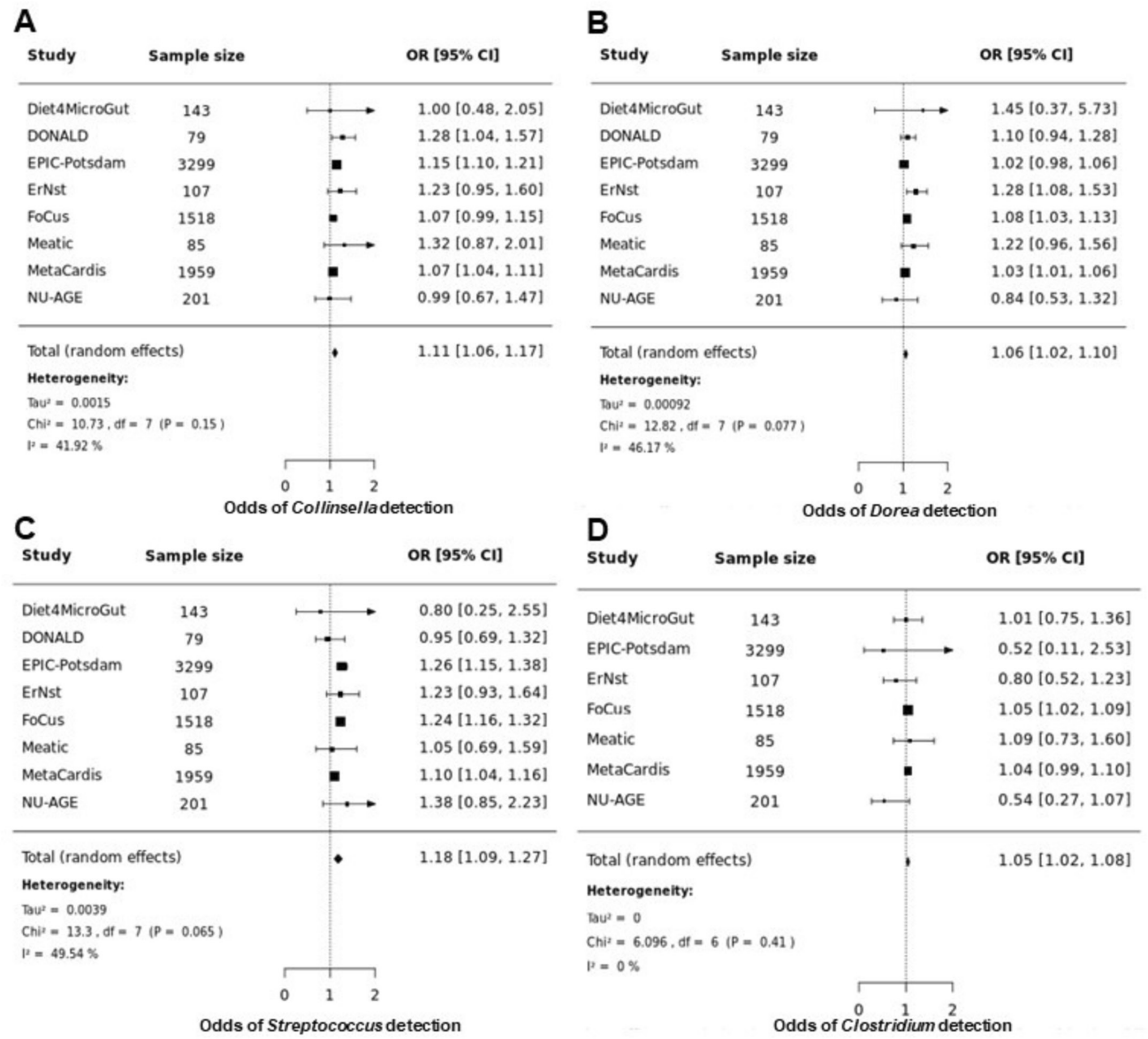
Forest plots of random‐effects study level meta‐analysis among adults from eight European studies, sex and age adjusted, showing odds of detection of (A) *Collinsella*, (B) *Dorea*, (C) *Streptococcus*, and (D) *Clostridium* per 5‐unit BMI increment.

The positive association between BMI and the phylum Proteobacteria observed in the linear regression was confirmed in the fractional regression, where a 5 kg/m^2^ higher BMI was associated with an 8% higher odds (OR 1.08, 95% CI: 1.04, 1.12, FDR‐adjusted *p* = 0.001) that a sequence read is assigned to Proteobacteria (Table 4). The inverse association between BMI and *Faecalibacterium* was also observed in the fractional regression analysis (OR 0.96, 95% CI: 0.92, 1.00, FDR‐adjusted *p* = 0.19). Although no association between BMI and *Roseburia* was observed in the linear regression, a positive association was observed in the fractional regression (OR: 1.04, 95% CI: 1.01, 1.06, FDR‐adjusted *p* = 0.01). Regarding less prevalent taxa, we observed a positive association between BMI and odds of *Collinsella* (Actinobacteria) detection (OR: 1.11, 95% CI: 1.06, 1.17, FDR‐adjusted *p* = 0.0003; Table 4 and Figure 3). Further, BMI was positively associated with odds of detection of *Clostridium* (OR: 1.05, 95% CI: 1.02, 1.08, FDR‐adjusted *p* = 0.02), *Dorea* (OR: 1.06, 95% CI: 1.02, 1.10, FDR‐adjusted *p* = 0.02), and *Streptococcus* (OR: 1.18, 95% CI: 1.09, 1.27; FDR‐adjusted *p* = 0.0002), three genera of the Firmicutes phylum (Table 4 and Figure 3).

**TABLE 4:** Study level meta‐analyses of fractional regressions on the associations of BMI (per 5‐unit increase) with odds of detection of taxa at the phylum and genus level.

Taxa	Prevalence^b^		OR (95% CI)^a^	Adjusted *p*‐value	Heterogeneity ChiSq	Heterogeneity *p*‐value	*I* ^2^ (%)
Phylum							
Actinobacteria	≥ 90%	7391	1.03 (1.00, 1.06)	0.10	6.04	0.53	8
Bacteroidetes	≥ 90%	7391	0.93 (0.83, 1.04)	0.38	119.82	< 0.0001	99
Firmicutes	≥ 90%	7391	1.02 (0.97, 1.08)	0.65	125.45	< 0.0001	96
Proteobacteria	≥ 90%	7391	1.08 (1.04, 1.12)	0.0007	14.46	0.04	48
Verrucomicrobia	10%–90%	7391	0.94 (0.87, 1.02)	0.29	13.48	0.06	38
Genus							
*Bifidobacterium* (Actinobacteria)	10%–90%	7391	1 (0.95, 1.06)	0.99	9.80	0.20	36
*Collinsella* (Actinobacteria)	10%–90%	7391	1.11 (1.06, 1.17)	0.0003	10.73	0.15	42
*Bacteroides* (Bacteroidetes)	≥ 90%	7391	0.99 (0.93, 1.04)	0.79	58.28	< 0.0001	87
*Odoribacter* (Bacteroidetes)	10%–90%	7391	0.93 (0.86, 1.00)	0.15	20.97	0.004	72
*Parabacteroides* (Bacteroidetes)	≥ 90%	7391	0.98 (0.93, 1.04)	0.76	31.12	0.0001	80
*Paraprevotella* ^*c*^ (Bacteroidetes)	10%–90%	7248	1 (0.92, 1.09)	0.99	11.37	0.08	41
*Prevotella* (Bacteroidetes)	10%–90%	7391	0.89 (0.65, 1.21)	0.67	23.69	0.001	97
*Blautia* (Firmicutes)	10%–90%	7391	1.09 (0.99, 1.19)	0.18	43.26	< 0.0001	83
*Clostridium* ^*d*^ (Firmicutes)	10%–90%	7312	1.05 (1.02, 1.08)	0.02	6.10	0.41	0
*Coprococcus* (Firmicutes)	10%–90%	7391	0.97 (0.95, 1.00)	0.09	3.78	0.80	0
*Dialister* (Firmicutes)	10%–90%	7391	1.06 (0.99, 1.13)	0.20	16.38	0.02	63
*Dorea* (Firmicutes)	10%–90%	7391	1.06 (1.02, 1.10)	0.02	12.82	0.08	46
*Eubacterium* (Firmicutes)	10%–90%	7391	1 (0.98, 1.03)	0.90	4.00	0.78	0
*Faecalibacterium* (Firmicutes)	≥ 90%	7391	0.96 (0.92, 1.00)	0.19	32.82	< 0.0001	77
*Lachnospira* (Firmicutes)^*e*^	10%–90%	5432	0.98 (0.9, 1.06)	0.74	6.75	0.34	15
*Oscillospira* (Firmicutes)^*f*^	10%–90%	3914	1 (0.94, 1.07)	0.99	5.88	0.32	0
*Roseburia* (Firmicutes)	≥ 90%	7391	1.04 (1.01, 1.06)	0.01	5.15	0.64	8
*Ruminococcus* (Firmicutes)	≥ 90%	7391	0.99 (0.94, 1.04)	0.80	26.76	0.0004	67
*Streptococcus* (Firmicutes)	10%–90%	7391	1.18 (1.09, 1.27)	0.0002	13.30	0.07	50
*Subdoligranulum* ^*c*^ (Firmicutes)	10%–90%	7248	0.99 (0.89, 1.10)	0.91	24.45	0.00	87
*Veillonella* (Firmicutes)^*g*^	10%–90%	7306	1.10 (1.01, 1.18)	0.09	7.10	0.31	0
*Enterobacter* ^*h*^ (Proteobacteria)	10%–90%	7163	0.57 (0.17, 1.95)	0.58	34.06	< 0.0001	99
*Sutterella* (Proteobacteria)	10%–90%	7391	1.10 (0.99, 1.23)	0.18	19.13	0.01	84
*Akkermansia* (Verrucomicrobia)	10%–90%	7391	0.95 (0.87, 1.03)	0.38	17.85	0.01	42

^a^ Fractional regression using generalized linear models (family quasibinomial, link logit), calculating odds ratios (ORs). Dependent variables: relative abundance proportions of taxa at the phylum/genus level; independent variables: BMI per 5 (kg/m^2^)‐unit increment plus sex and age (years) for adjustment.

^b^ Detailed study‐specific taxa prevalences are displayed in Table S4.

^c^ Model for *Subdoligranum* and *Paraprevotella* exclude Diet4MicroGut (data not available).

^d^ Model for *Clostridium* excludes DONALD (data not available).

^e^ Model for *Lachnospira* excludes MetaCardis (data not available).

^f^ Model for *Oscillospira* excludes FoCus and MetaCardis (data not available).

^g^ Model for *Veillonella* excludes MeaTIc (data not available).

^h^ Model for *Enterobacter* excludes Diet4MicroGut and MeaTIc (data not available).

### 3.6 Sensitivity Analysis: Adjustment for Fiber Intake

This analysis excluded the EPIC‐Potsdam substudy and MetaCardis, as fiber intake data were not available. The association between higher BMI and lower Shannon index (*β*: −0.04, 95% CI: −0.06, −0.03) persisted after exclusion of these two studies and was not changed by adjustment for energy‐adjusted fiber residuals and energy intake (*β*: −0.04, 95% CI: −0.05, −0.03). In addition, the associations between higher BMI and higher odds of detection of *Collinsella*, *Dorea*, and *Streptococcus* persisted after exclusion of the EPIC‐Potsdam substudy and MetaCardis (OR [95% CI] 1.14 [1.02, 1.26], 1.11 [1.04, 1.20], 1.22 [1.15, 1.30], respectively) and remained unchanged after adjustment for fiber residuals with energy intake adjustment (OR [95% CI] 1.09 [1.01, 1.18], 1.10 [1.04, 1.17], 1.16 [1.02, 1.33], respectively). All other observed associations were slightly attenuated after exclusion of the EPIC‐Potsdam substudy and MetaCardis, and these associations were not affected by fiber adjustment (data not shown).

## 4 Discussion

In this individual‐level data meta‐analysis of cross‐sectional study data, gathering eight European observational studies, we found consistent evidence that higher BMI was associated with lower alpha diversity (Shannon index). Further, BMI was positively associated with the phylum Proteobacteria, but no associations were found for the phyla Firmicutes and Bacteroidetes or with the *F*:*B* ratio, with high between‐study heterogeneity. On the genus level, higher BMI was associated with lower relative abundance of *Faecalibacterium* of the Firmicutes phylum but with higher odds of detection of *Dorea*, *Clostridium*, and *Streptococcus*, three genera also belonging to Firmicutes. Further, a higher odds of detection of *Collinsella* of the Actinobacteria phylum was observed with higher BMI.

A literature‐based systematic review summarizing the evidence on the association between obesity and gut microbiota composition revealed that out of 22 studies, nine found an inverse association with the Shannon index, whereas 11 found no statistically significant association, and two found a positive association. Further, in the meta‐analysis of seven studies, there was between‐study heterogeneity of 83%, and the combined estimate indicated a weak inverse association [9]. Our current study extends these findings by supporting an inverse association of BMI with the Shannon index. Despite observing substantial heterogeneity in our study as well (60%), effect estimates for every participating study pointed to an inverse association.

In our study, we neither found evidence of a positive association of BMI with the *F*:*B* ratio, nor with Firmicutes relative abundance, nor an inverse association with Bacteroidetes relative abundance. Our findings for the *F*:*B* ratio were consistent with the previous meta‐analysis [9], where no overall association was found in people with versus without obesity. However, higher Firmicutes relative abundance comparing people with versus without obesity was observed in a meta‐analysis of six studies. Both the present study and the literature‐based meta‐analysis [9] show very high heterogeneity for the analysis with Firmicutes and Bacteroidetes, which may be explained by unaccounted differences between studies and study populations, as well as study‐specific differences in the composition of genera or species belonging to Firmicutes. In the present study, we also observed no association between BMI and *P*:*B* ratio, although previous studies reported a higher *P*:*B* ratio among participants with obesity [55] or higher *Prevotella* relative abundance [23, 56, 57] and lower Bacteroides relative abundance [18, 58] comparing people with versus without obesity. In the previous systematic review, three studies reported higher *Prevotella* abundance in people with versus without obesity, whereas in the case of Bacteroides, results were inconsistent (three studies reported higher and two studies lower Bacteroides in people with vs. without obesity) [9].

In line with a systematic review reporting a consistent association of Proteobacteria with obesity [59], we observed a positive association of BMI with the relative abundance as well as the odds of detection of Proteobacteria with substantial heterogeneity. On the study level, a positive association was observed in all but two smaller population‐based studies (ErNst and NU‐Age) that do not differ substantially regarding main characteristics from the rest of the studies, although ErNst had a relatively low obesity prevalence, whereas NU‐Age is the study with the highest average age. The phylum Proteobacteria has been associated with inflammation and microbial dysbiosis [60] and could play a role in obesity‐associated disease risk. However, *Proteobacteria* were not investigated in detail in the present study (only *Enterobacter* and *Sutterella*, which showed no association in the fractional regression analysis), so further research on the genus and species level could provide insights into the specific roles of *Proteobacteria* in obesity.

On the genus level, our findings of an inverse association of BMI and relative abundance of *Faecalibacterium* (with low heterogeneity) support findings from some previous studies [58, 61], but not those of others [18, 57]. A depletion in the fiber‐degrading, short‐chain fatty acid‐producing *Faecalibacterium* has been observed in nonalcoholic steatohepatitis (NASH), the more severe form of nonalcoholic fatty liver disease (NAFLD), which is accompanied by inflammation and oxidative stress [62], pointing to a potential disease‐protective role of *Faecalibacterium* in obesity.

In contrast, we observed positive BMI associations with the odds of detection of three genera of the Firmicutes phylum (*Streptococcus*, *Dorea*, and *Clostridium*). The positive association between BMI and *Streptococcus* was observed with moderate heterogeneity, i.e., a positive association was observed in all but two population‐based studies, i.e., Diet4MicroGut and DONALD, that both had a lower mean age compared with the other studies, a majority of participants with normal weight, and no prevalent cardiometabolic diseases in Diet4MicroGut and a low prevalence presumably also in DONALD. Our findings of a positive association between BMI and *Streptococcus* were consistent with the systematic review by Pinart et al. (five studies found significantly higher relative abundance comparing people with vs. without obesity) [9]. *Streptococcus* enrichment has been observed among people with obesity, comparing those with versus without NAFLD, suggesting a potential role in obesity‐associated comorbidity [63].

Higher BMI was associated with a higher odds of *Dorea* detection with moderate heterogeneity: A positive association was observed in all studies except NU‐AGE, which is the study with the oldest study population, but the prevalence of cardiometabolic diseases was not available for this study. In addition, higher BMI was positively associated with the odds of detection of *Clostridium*, with low heterogeneity, although inverse associations were observed in NU‐AGE and EPIC‐Potsdam, the detection proportions in these two studies were close to zero. Both *Dorea* and *Clostridium* have been previously found to be enriched in people with obesity [9] and have been related to increased gut permeability and inflammation and may therefore play a functional role for obesity‐related chronic disease risk [64].

The observed positive association between BMI and odds of detection of the genus *Collinsella*, of the phylum Actinobacteria, is supported by previously observed enrichment in people with obesity [65]. *Collinsella* enrichment has also been observed in Type 2 diabetes [66] and rheumatoid arthritis [67], suggesting a potential pro‐inflammatory role (e.g., relative abundance of *Collinsella* correlated with the proinflammatory cytokine IL‐17A), coupled with experimental evidence that *Collinsella* may increase gut permeability [67]. Furthermore, low fiber intake has been positively associated with *Collinsella* abundance in the gut microbiota of overweight and pregnant women with obesity [68]. However, in our study, the positive association between BMI and the odds of detection of *Collinsella* was only slightly attenuated by fiber adjustment. More human research into how *Collinsella* influences host metabolism and/or gut permeability in people with and without obesity and the role of dietary intake is warranted.

Other previously observed associations at the genus level were not confirmed by the present study; for example, we did not find consistent associations of BMI with *Dialister*, *Eubacterium*, *Lachnospira*, *Roseburia*, and *Ruminococcus*, which have been reported to be higher among people with obesity [9]. We also did not find associations of BMI with *Akkermansia*, *Paraprevotella*, or *Subdoligranulum*, which have been found in other studies to be inversely associated with obesity [9].

We found no evidence of an impact of dietary fiber intake adjustment on the association between BMI and Shannon index and odds of detection of *Collinsella*, *Dorea*, and *Streptococcus*, suggesting that these associations cannot be explained by variation in fiber intake. However, these were only sensitivity analyses, and information on dietary intake was not available in the two largest studies included in this analysis. Therefore, a deeper analysis of the role of potential interaction by fiber intake was beyond the scope of this study. Fiber intake was weakly inversely correlated with BMI in most studies. Of the few previous investigations on the role of fiber intake as a potential confounder, one small study observed that fiber adjustment did not attenuate the observed associations between BMI and microbiota diversity and composition [69]. In another study, the association between BMI categories and taxa belonging to the Firmicutes and Actinobacteria phyla was attenuated after adjustment for dietary fiber [70], although the association with the family *Paraprevotellaceae* (phylum *Bacteroidetes*) persisted. Although dietary fibers directly interact with the gut microbiota [71], several observational studies did not report an association between fiber intake and microbiota composition [21, 23, 72, 73], which may explain why we did not observe an impact on the association of BMI with gut microbiota composition after fiber intake adjustment. In addition, measurement errors in estimating fiber intake could also have played a role [74].

Some explanations for the discrepant results found in the literature on the associations of BMI with gut microbiota composition include differences in the populations, such as sex, age, prevalence of chronic diseases, geographical region, and diet. Although we used harmonized variables in our study, the harmonization procedure was retrospective. Retrospective harmonization can improve data comparability across studies but is still subject to background noise from different data collection methods [75]. More specifically, one important source of heterogeneity that can lead to discrepant results is related to differences in DNA extraction methods, sequencing, and data processing techniques [76]. In our study, the most common method used to determine microbiota composition was sequencing of the variable region V3–V4 within the 16S rRNA gene, but three studies sequenced other 16S rRNA gene regions, and one study used shotgun metagenomics. Additionally, different taxonomic databases were used. Both the database and the targeted sequencing region can influence genera annotation and thereby can ultimately affect the comparability across studies. If raw data are available locally, these differences can be addressed to some extent, but complete standardization remains challenging. Our observation of consistent results in terms of direction, for example, for the association between BMI and Shannon index, however, demonstrates the value of comparison of studies with slightly different microbiota assessment techniques.

This study has several strengths, including the large sample from eight studies from several European countries (Italy, Germany, France, and Denmark) and the high diversity of the study population, including a broad age range (mean age ranging from 25 to 71 years). Although several studies have addressed the research question of body fatness and gut microbiota composition, inconsistent results in human studies [9] highlight the importance of examining this question in a large and diverse population with harmonized data. In the present meta‐analysis, the GLMs for every single study included the same harmonized variables, overcoming some important limitations identified in the previous systematic review, i.e., different definitions of exposure and covariables, as well as a general lack of adjustment even for major confounders such as age and sex [9]. Another strength is the federated data environment through DataSHIELD. This method facilitated the analysis of data from multiple studies without sharing and pooling data; this reduces the administrative burden and ethical challenges associated with data sharing, as well as a reduced burden on analytical capacity in individual studies (as compared with alternative forms of data sharing), all of which may be a barrier in collaborative projects [28]. Additionally, we were able to compare results with two different approaches: two‐stage (SLMA) and one‐stage IPD analysis; a previous study found both approaches to provide consistent results, but more conservative results in two‐stage IPD [44]. We additionally found out that exceptions where results differed were characterized by high between‐study heterogeneity, providing valuable information to consider about one‐stage IPD analysis validity in specific cases and highlighting the importance of taking heterogeneity into consideration when interpreting results.

Our study also has some limitations, for example, the cross‐sectional nature of our analysis precludes any insight into direction and causality, and residual confounding cannot be ruled out. Another limitation deals with differences in data collection methods across studies, including the sequenced genetic marker and taxonomic database used. We did not use specific statistical packages for microbiome data (such as Maaslin2) because these were not available for DataSHIELD at the time of analysis. However, we tested different statistical approaches based on one study (MetaCardis) and found highly consistent results in terms of direction of association and statistical significance when comparing beta regression (continuous part of model only), Maaslin2 package (continuous part of model only) [77], ZOIB package (continuous and binary part of model), and our approach (continuous and binary model) [78], strengthening the confidence in our statistical approach in a federated analysis of multiple studies. In addition, we found largely consistent results from the linear and fractional regressions for prevalent taxa. Finally, our results are not generalizable to the European population since the included studies were all limited to the INTIMIC‐KP consortium, and the study participants were not in all studies selected from the general population.

## 5 Conclusion

Our results provide evidence of an inverse association between BMI and alpha diversity as well as a positive association with Proteobacteria, whereas we found no association with Firmicutes, Bacteroidetes, or the *F*:*B* ratio and observed high between‐study heterogeneity in these associations. On the genus level, we observed inverse associations of BMI with *Faecalibacterium*, and positive associations with *Dorea*, *Streptococcus*, *Clostridium* (all Firmicutes), and *Collinsella* (Actinobacteria). By including a large and diverse study population from eight European studies, our findings offer valuable insights into the association of BMI with gut microbiota composition. Further efforts toward the harmonization of data, especially microbiota data processing methods, could help address the high between‐study heterogeneity and explain the highly inconsistent results reported in the literature. Such analysis could also be conducted in a federated data environment, which may increase the willingness of studies to contribute to such a comprehensive analysis by eliminating the need to share and pool data directly.

## Author Contributions

C.S., M.P., K.N., and T.P. were responsible for the study conception and design. Instructions for dataset preparation, data harmonization, and data upload in Opal for the participating studies were developed by S.M.S., M.P., C.S., and K.N. Data harmonization, preparation, and analysis for this federated analysis were made possible with the support of the following coauthors: C.D.F., F.D.F., and D.E. (Diet4Microgut); U.N. and K.O. (DONALD); M.B.S. and F.S. (EPIC‐Potsdam substudy); a.d. (ErNst); M.L. and K.S. (FoCus); F.M.C. and M.D.A. (MeaTic); S.K.F.‐S. (MetaCardis); and S.T., P.B., and M.F. (NU‐AGE). Data analyses were performed by C.S., S.M.S., F.S. and K.N. All coauthors contributed to the interpretation of the data. The first draft of the manuscript was written by C.S., and all authors commented on previous versions of the manuscript critically for important intellectual content. All authors read and approved the final manuscript. K.N. and T.P. had primary responsibility for final content.

## Funding

This research was supported by the Joint Action “European Joint Programming Initiative “A Healthy Diet for a Healthy Life” (JPI HDHL)” and the respective national/regional funding organizations: Fund for Scientific Research (FRS—FNRS, Belgium); Research Foundation—Flanders (FWO, Belgium); INSERM Institut National de la Santé et de la Recherche Médicale (France); Bundesministerium für Ernährung und Landwirtschaft (BMEL) represented by Federal Office for Agriculture and Food (BLE, Germany); Bundesministerium für Bildung und Forschung (BMBF, FKZ 01EA1906A, 01EA1906B, 01EA1906F); Ministero dell';Istruzione, dell'Università e della Ricerca (MIUR), Ministry of Agricultural, Food and Forestry Policies (MiPAAF), National Institute of Health (ISS) on behalf of Ministry of Health (Italy); National Institute of Health Carlos III (Spain); the Netherlands Organization for Health Research and Development (ZonMw, the Netherlands); Austrian Research Promotion Agency (FFG) on behalf of the Bundesministerium für Bildung, Wissenschaft, Forschung und Technologie (BMBWF); Ministry of Science and Technology (Israel); Formas (Sweden) and HDHL‐Intimic Era‐Net (EarlyFOOD); Joint Programming Initiative a Healthy Diet for a Healthy Life‐Intestinal Microbiomics (JPI HDHL‐INTIMIC) Call for Joint Transnational Research Proposals on “Interrelation of the Intestinal Microbiome, Diet and Health” (Reference Number JTC‐2017‐7); National Recovery and Resilience Plan (NRRP), Mission 4 Component 2 Investment 1.3—Call for tender No. 341 of March 15, 2022, of Italian Ministry of University and Research funded by the European Union—NextGenerationEU; Award Number: Project code PE00000003, Concession Decree No. 1550 of October 11, 2022, adopted by the Italian Ministry of University and Research, Project title “ON Foods ‐ Research and innovation network on food and nutrition Sustainability, Safety and Security – Working ON Foods”; the EPIC Potsdam substudy was supported by a Grant from the Ministerium für Ländliche Entwicklung, Umwelt und Landwirtschaft des Landes Brandenburg (DZD Grant 82DZD00302). The implementation of DataSHIELD was supported with funding from the National Research Data Infrastructure for Personal Health Data (NFDI4Health) by the Deutsche Forschungsgemeinschaft (DFG, German Research Foundation)—Project number 442326535. The positions of the authors Carolina Schwedhelm, Sofia M. Siampani, and Florian Schwarz were funded by NFDI4Health. In light of the extensive preparation of the dataset and scripting necessary to conduct the here described DataSHIELD analyses, two DataSHIELD packages have been developed with the support of NFDI4Health that focus on (a) improved user experience and client‐side driven calculation of robust standard errors (dsSupportClient) and (b) a simpler pathway to calculate GLMs for log‐transformed, zero‐inflated data (dsIntestinalMicrobiomics).

## Conflicts of Interest

C.S. was an employee of Pfizer in Germany at the time of article submission. None of the other authors reported any conflicts of interest.

## Supporting information


**TABLE S1:** Harmonized variables available for the federated meta‐analysis of eight European studies.
**TABLE S2:** Spearman correlation between dietary fiber intake and body mass index in participants from the included studies with dietary intake data.
**TABLE S3:** Baseline characteristics of the participants from the included studies, by BMI category.
**TABLE S4:** Taxa prevalence and distribution in samples across eight European studies (N = 7415).
**TABLE S5:** Individual person data analyses of the associations between BMI (per 5‐unit increase) and alpha diversity, relative abundance of prevalent taxa on the phylum and genus levela and F:B and P:B ratios.
**FIGURE S1:** Histogram of log‐transformed Prevotella to Bacteroides ratio among adults from eight European studies.
**FIGURE S2:** Forest plots of random‐effects study‐level meta‐analysis among adults from eight European studies, sex and age adjusted, showing the mean difference in relative abundance of prevalent taxa (present in ≥ 90% of samples) on the phylum level per 5‐unit BMI increment. Phyla are (A) Firmicutes, (B) Bacteroidetes, (C) Actinobacteria (log‐transformed), and (D) Proteobacteria (log‐transformed).
**FIGURE S3:** Forest plots of random‐effects study‐level meta‐analysis among adults from eight European studies, sex and age adjusted, showing the mean difference in log‐transformed relative abundance of prevalent taxa (present in ≥ 90% of samples) on the genus level per 5‐unit BMI increment. Genera are (A) Bacteroides, (B) Proteobacteria, (C) Faecalibacterium, (D) Roseburia, and (E) Ruminococcus.

## Data Availability

The data underlying this article are not openly available due to reasons of sensitivity. The informed consent of the study participants does cover uploading data (also not anonymized) to public repositories. Harmonized datasets comprising microbiota data as well as phenotypic variables were uploaded to local Opal servers by the study‐specific data holders for federated analysis. The data can be accessed via DataSHIELD, a software enabling privacy‐preserving individual‐level data query and analysis with controlled access. The metadata of the uploaded data is published here: https://mica.mdc‐berlin.de/. Researchers can query the metadata and request federated data access via DataSHIELD by submitting a request as outlined here (https://mica.mdc‐berlin.de/) via e‐mail: epidemiologie@mdc-berlin.de. Federated access to the data via DataSHIELD can be granted to researchers after approval of their request by the data holders.
