# Supplementary material for: Associations of Adiposity With Gut Microbiota Composition Among Adults—Results From a Federated Analysis of Individual Participant Data From Eight European Observational Studies
Source: Obes Rev. 2026 Mar 1;27(8):e70106. doi: 10.1111/obr.70106 (PMC13371411; doi:10.1111/obr.70106)
Supplement: Supplementary file 1 — TABLE S1: Harmonized variables available for the federated meta‐analysis of eight European studies. TABLE S2: Spearman correlation between dietary fiber intake and body mass index in participants from the included studies with dietary intake data. TABLE S3: Baseline characteristics of the participants from the included studies, by BMI category. TABLE S4: Taxa prevalence and distribution in samples across eight European studies (N = 7415). TABLE S5: Individual person data analyses of the associations between BMI (per 5‐unit increase) and alpha diversity, relative abundance of prevalent taxa on the phylum and genus levela and F:B and P:B ratios. FIGURE S1: Histogram of log‐transformed Prevotella to Bacteroides ratio among adults from eight European studies. FIGURE S2: Forest plots of random‐effects study‐level meta‐analysis among adults from eight European studies, sex and age adjusted, showing the mean difference in relative abundance of prevalent taxa (present in ≥ 90% of samples) on the phylum level per 5‐unit BMI increment. Phyla are (A) Firmicutes, (B) Bacteroidetes, (C) Actinobacteria (log‐transformed), and (D) Proteobacteria (log‐transformed). FIGURE S3: Forest plots of random‐effects study‐level meta‐analysis among adults from eight European studies, sex and age adjusted, showing the mean difference in log‐transformed relative abundance of prevalent taxa (present in ≥ 90% of samples) on the genus level per 5‐unit BMI increment. Genera are (A) Bacteroides, (B) Proteobacteria, (C) Faecalibacterium, (D) Roseburia, and (E) Ruminococcus. [file OBR-27-e70106-s001.docx]

**TITLE:** Associations of adiposity with gut microbiota composition among adults – results from a federated analysis of individual participant data from eight European observational studies

**AUTHORS:** Carolina Schwedhelm^1^, Mariona Pinart^1^, Sofia K Forslund-Startceva^2,3,4,5,6^, Kolade Oluwagbemigun^7^, Andreas Dötsch^8^, Kristina Schlicht^9^, Florian Schwarz^10^, Sofia M Siampani^1^, Demetris Avraam^11^, Maria De Angelis^12^, Jildau Bouwman^13^, Patrizia Brigidi^14^, Giovanna Caderni^15^, Francesco Maria Calabrese^12^, Rafael RC Cuadrat^10^, Carlotta De Filippo^16^, Francesca De Filippis^17,18^, Danilo Ercolini^17,18^, Marco Fabbrini^14,19^, Matthias Laudes^9^, Ute Nöthlings^7^, Serdar Özsezen^13^, Itai Sharon^20,21^, Matthias B Schulze^10,22^, Silvia Turroni^19^, Francesco Vitali^16^, Tobias Pischon^1,3,23^, Katharina Nimptsch^1^

**AFFILIATIONS:**

1. Max Delbrück Center for Molecular Medicine in the Helmholtz Association (MDC), Molecular Epidemiology Research Group, Berlin, Germany
2. Experimental and Clinical Research Center, A Cooperation of Charité-Universitätsmedizin Berlin and Max Delbrück Center for Molecular Medicine in the Helmholtz Association (MDC), Berlin, Germany
3. Charité-Universitätsmedizin Berlin, Corporate Member of Freie Universität Berlin, Humboldt-Universität zu Berlin, Berlin Institute of Health, Berlin, Germany
4. Host-Microbiome Factors in Cardiovascular Disease Lab, Max Delbrück Center for Molecular Medicine in the Helmholtz Association (MDC), Berlin, Germany
5. Structural and Computational Biology Unit, European Molecular Biology Laboratory, Heidelberg, Germany
6. German Centre for Cardiovascular Research (DZHK), Partner Site Berlin, Berlin, Germany
7. Department of Nutrition and Food Sciences, Nutritional Epidemiology, University of Bonn, Bonn, Germany
8. Department of Physiology and Biochemistry of Nutrition, Max Rubner-Institut (MRI) – Federal Research Institute of Nutrition and Food, Karlsruhe, Germany
9. Institute of Diabetes and Clinical Metabolic Research, University of Kiel, Kiel, Germany
10. Department of Molecular Epidemiology, German Institute of Human Nutrition Potsdam-Rehbruecke, Nuthetal, Germany
11. Section of Epidemiology, Department of Public Health, University of Copenhagen, Copenhagen, Denmark
12. Department of Soil Plant and Food Sciences, University of Bari Aldo Moro, Bari, Italy
13. Microbiology and Systems Biology Group, Toegepast Natuurwetenschappelijk Onderzoek (TNO), Leiden, The Netherlands
14. Microbiomics Unit, Department of Medical and Surgical Sciences, University of Bologna, Bologna, Italy
15. NEUROFARBA Department, Pharmacology and Toxicology Section, University of Florence, Florence, Italy
16. Institute of Agricultural Biology and Biotechnology National Research Council, Pisa, Italy
17. Department of Agricultural Sciences, University of Naples Federico II, Portici, Italy
18. Task Force on Microbiome Studies, University of Naples Federico II, Naples, Italy
19. Unit of Microbiome Science and Biotechnology, Department of Pharmacy and Biotechnology, University of Bologna, Bologna, Italy
20. Migal-Galilee Research Institute, Kiryat Shmona, Israel
21. Faculty of Sciences and Techology, Tel-Hai Academic College, Upper Galilee, Israel
22. Institute of Nutritional Science, University of Potsdam, Nuthetal, Germany
23. Biobank Technology Platform, Max-Delbrueck-Center for Molecular Medicine in the Helmholtz Association (MDC), Berlin, Germany

**Corresponding author:**

Dr. Katharina Nimptsch

Molecular Epidemiology Research Group
Max Delbrück Center for Molecular Medicine in the Helmholtz Association (MDC)
Robert-Rössle-Straße 10
13125 Berlin-Buch, Germany

Telephone:+49 30 9406-1933
Fax: +49 30 9406-4576

E-Mail: [Katharina.nimptsch@mdc-berlin.de](mailto:Katharina.nimptsch@mdc-berlin.de)

ORCID ID: 0000-0001-7877-205X

SUPPLEMENTAL METHODS:

**Details on study-specific ethical approvals:**

Diet4MicroGut was approved by the Ethics Committee of (a) Azienda Sanitaria Locale (Bari) (protocol N.1050), (b) Azienda Ospedaliera Universitaria of Bologna (protocol N.0018396), (c) Province of Parma (protocol N.22884) and (d) University of Torino (protocol N.1/2013/C).

DONALD was approved by the Scientific Committee of the Research Institute of Child Nutrition.

EPIC-Potsdam substudy was approved by the Ethical Committee of the State of Brandenburg, Germany (S9/2002).

ErNst was approved by the Ethics Committee of the State Medical Chamber of Baden-Württemberg (EK LÄK BW; F-2018-055) in October 2018.

FoCus was approved by the local ethics committee of the Kiel University (A156-03/Date 2011/07/28).

Meatic was approved by the Tuscany Regional Ethics Committee of the University Hospital of Careggi, Florence (SPE, #12390).

MetaCardis was approved by Ethics Committee CPP Ile-de France, Ethics Committee at the Medical Faculty at the University of Leipzig, and the Ethical Committees of the Capital Region of Denmark.

NU-AGE was approved by the ethics committee of the coordinator center (the Independent Ethics Committee of the S. Orsola-Malpighi Hospital Bologna (Italy)) and by the local/national ethics committees of all the other four recruiting centers.

**Local data harmonization process**

A data catalogue with the names, descriptions, units or categories, and value format of the harmonized variables was circulated among the participating studies. Harmonized variables included besides the exposure (BMI) and outcome variables (Shannon Index, relative abundances at the phylum and genus level) age in years, sex (male/female), smoking status (never/former/current), school education (less than 12 years/12 years or more) and further variables related to prevalent diseases, medication, probiotics use, and dietary intake variables. Each study harmonized their data to match the data catalogue; if studies could not provide some variables, those variables were omitted (missing) and the rest of the dataset was prepared. A list of the requested variables and availability per study is available in **Supplemental Table 1**. Study partners uploaded the harmonized datasets to their local servers together with the corresponding data dictionaries.

**Centralized data harmonization**

In some studies, further necessary data harmonization steps based on the uploaded data were performed using DataSHIELD: 1) the EPIC-Potsdam sub-study provided two smoking variables, one according to the definition of the harmonized variable but missing in 34 individuals, and a second smoking variable available in all participants with microbiota data, but with different response categories (smoked in the past 24h or seven days prior to the stool sample: yes/no). To obtain the harmonized variable, missing values in the first smoking variable were classified as 3=current smoker if the response in the second variable was “yes”. 2) In FoCus, energy intake (kcal/day) was derived from macronutrient and alcohol intake (g/day) using 4 kcal/g for carbohydrates and protein, 9 kcal/g of fat, and 7 kcal/g of alcohol. 3) Also, in FoCus, N=14 with an uninterpretable category in school education were assigned to a missing category for this variable.

The variable “prevalent cardiometabolic diseases” was derived by combining prevalent hypertension, diabetes, cardiovascular diseases and dyslipidemia (i.e., set to “1” if any individual disease variable was present). The current use of medication was derived by combining diabetes medication, statins, and nonsteroidal anti-inflammatory drug usage (i.e., set to “1” if any individual medication variable was taken).

F:B ratio was calculated by adding a pseudocount of 0.01% to both relative abundances of *Firmicutes* (a_F_) and *Bacteroidetes* (**a_B_**) and calculating a_F_/a_B_.. Similarly, (*P:B*) ratio was calculated by dividing both taxa’s abundances after adding a pseudocount of 0.01%.

## SUPPLEMENTAL TABLE 1: Harmonized variables available for the federated meta-analysis of eight European studies

| **Harmonized variable name** | **Description** | **Units or categories** |
| --- | --- | --- |
| ***Sociodemographic & lifestyle variables*** | | |
| AGE | Age | years |
| SEX | Sex | 1=male  2=female |
| SMOKE_ST | Smoking status | 1=never smoker  2=former smoker  3=current smoker |
| EDU | School education | 1=less than 12 years  2=12 or more years |
| PREV_HYP | Prevalent hypertension | 1=yes  0=no |
| PREV_DIAB | Prevalent diabetes | 1=yes  0=no |
| PREV_CVD | Prevalent cardiovascular diseases | 1=yes  0=no |
| PREV_DYS | Prevalent dyslipidemia | 1=yes  0=no |
| REG_PREBIOTICS | Regular use of probiotics | 1=yes  0=no |
| MED_DIAB | Current use of diabetes medication | 1=yes  0=no |
| MED_STAT | Current use of statins | 1=yes  0=no |
| MED_NSAID | Current use of nonsteroidal anti-inflammatory drugs | 1=yes  0=no |
| PAST_AB | Use of antibiotics in the past months | 1=yes  0=no |
| PAST_AB_TIME | Time period for “Use of antibiotics in the past months” | 1=in the past 0-3 months  2=in the past 4-6 months  3=in the past 7-12 months  4=in the past 12+ months |
| ***Anthropometric variables*** | | |
| WEIGHT | Body weight | kg |
| HEIGHT | Height | cm |
| ***Dietary intake variables*** | | |
| DIET_M | Dietary assessment method | 1=24-hour recall, 2=FFQ, 3=Food record, 4=other |
| MMYY_DIET | Month of dietary assessment | months |
| ENERGY | Energy intake | kcal/day |
| CARB | Carbohydrate intake | g/day |
| FIBER | Fiber intake | g/day |
| FAT | Total fat intake | g/day |
| SFA | Saturated fatty acid intake | g/day |
| MUFA | Monounsaturated fatty acid intake | g/day |
| PUFA | Polyunsaturated fatty acid intake | g/day |
| PROT | Protein intake | g/day |
| ALC | Alcohol intake | g/day |
| ***Gut microbiome variables*** | | |
| P_ACTINOBACTERIA | relative abundance of phylum actinobacteria | % |
| P_BACTEROIDETES | relative abundance of phylum bacteroidetes | % |
| P_FIRMICUTES | relative abundance of phylum firmucutes | % |
| P_PROTEOBACTERIA | relative abundance of phylum proteobacteria | % |
| P_VERRUCOMICROBIA | relative abundance of phylum verrucomicrobia | % |
| G_BIFIDOBACTERIUM | relative abundance of genus bifidobacterium | % |
| G_COLLINSELLA | relative abundance of genus collinsella | % |
| G_BACTEROIDES | relative abundance of genus bacteroides | % |
| G_ODORIBACTER | relative abundance of genus odoribacter | % |
| G_PARABACTEROIDES | relative abundance of genus parabacteroides | % |
| G_PARAPREVOTELLA | relative abundance of genus paraprevotella | % |
| G_PREVOTELLA | relative abundance of genus prevotella | % |
| G_RIKENELLA | relative abundance of genus rikenella | % |
| G_BLAUTIA | relative abundance of genus blautia | % |
| G_CLOSTRIDIUM | relative abundance of genus clostridium | % |
| G_COPROCOCCUS | relative abundance of genus coprococcus | % |
| G_DIALISTER | relative abundance of genus dialister | % |
| G_DOREA | relative abundance of genus dorea | % |
| G_EUBACTERIUM | relative abundance of genus eubacterium | % |
| G_FAECALIBACTERIUM | relative abundance of genus faecalibacterium | % |
| G_LACHNOSPIRA | relative abundance of genus lachnospira | % |
| G_OSCILLOSPIRA | relative abundance of genus oscillospira | % |
| G_ROSEBURIA | relative abundance of genus roseburia | % |
| G_RUMINOCOCCUS | relative abundance of genus ruminococcus | % |
| G_STREPTOCOCCUS | relative abundance of genus streptococcus | % |
| G_SUBDOLIGRANULUM | relative abundance of genus subdoligranulum | % |
| G_VEILLONELLA | relative abundance of genus veillonella | % |
| G_ENTEROBACTER | relative abundance of genus enterobacter | % |
| G_SUTTERELLA | relative abundance of genus sutterella | % |
| G_AKKERMANSIA | relative abundance of genus akkermansia | % |
| S_W_INDEX | Shannon-Wiener alpha diversity index |  |

SUPPLEMENTAL TABLE 2: Spearman correlation between dietary fiber intake and body mass index in participants from the included studies with dietary intake data

|  |  | **Spearman correlation between BMI and total fiber intake** | | **Spearman correlation between BMI and energy-adjusted fiber residuals** | |
| --- | --- | --- | --- | --- | --- |
|  | **N** | **r** | **p-value** | **r** | **p-value** |
| Diet4MicroGut | 143 | 0.12 | 0.16 | -0.01 | 0.90 |
| DONALD | 79 | 0.18 | 0.11 | 0.06 | 0.59 |
| ErNst | 107 | -0.05 | 0.61 | -0.24 | 0.01 |
| FoCus | 1443 | -0.05 | 0.04 | -0.14 | <0.0001 |
| MeaTIc | 85 | -0.02 | 0.89 | -0.04 | 0.70 |
| NU-AGE | 201 | -0.11 | 0.11 | -0.08 | 0.24 |

SUPPLEMENTAL TABLE 3: Baseline characteristics of the participants from the included studies^a^, by BMI category

| Characteristics | Diet4MicroGut | | | | DONALD | | | EPIC-Potsdam substudy | | | ErNst | | | FoCus | | | MeaTIc | | | MetaCardis | | | NU-AGE | | |
| --- | --- | --- | --- | --- | --- | --- | --- | --- | --- | --- | --- | --- | --- | --- | --- | --- | --- | --- | --- | --- | --- | --- | --- | --- | --- |
| *BMI (kg/m^2^)* | <25 | | 25 to > 30 | > 30 | <25 | 25 to > 30 | > 30 | <25 | 25 to > 30 | > 30 | <25 | 25 to > 30 | > 30 | <25 | 25 to > 30 | > 30 | <25 | 25 to > 30 | > 30 | <25 | 25 to > 30 | > 30 | <25 | 25 to > 30 | > 30 |
| N | 125 | | 18 | 0 | 57 | 14 | 8 | 1164 | 1374 | 761 | 65 | 33 | 9 | 507 | 418 | 595 | 50 | 26 | 9 | 430 | 504 | 1025 | 61 | 102 | 37 |
| Sociodemographic | |  |  |  |  |  |  |  |  |  |  |  |  |  |  |  |  |  |  |  |  |  |  |  |  |
| Sex, female | 80  (64) | | 3  (16.7) | - | 40  (70.2) | 8  (57.1) | 3  (37.5) | 810 (69.6) | 683 (49.7) | 414 (54.4) | 37  (56.9) | 10 (30.3) | 6  (66.7) | 354 (69.8) | 185 (44.3) | 418 (70.3) | 38  (76) | 16 (61.5) | 6  (66.7) | 223 (51.9) | 139 (27.6) | 613 (59.8) | 34 (55.7) | 47 (46.1) | 19 (51.4) |
| Age, y | 38.16 ± 9.63 | | 37.11 ± 8.15 | - | 23.98 ± 5.56 | 27.64 ± 6.79 | 27.88 ± 6.96 | 66.97 ± 8.38 | 69.37 ± 7.89 | 68.78 ± 7.79 | 47.55 ± 17.30 | 48.70 ± 17.41 | 62.33 ± 12.99 | 50.62 ± 15.46 | 55.4 ± 13.63 | 50.04 ± 13 | 34.44 ± 9.75 | 38.73 ± 10.74 | 47.67 ± 5.59 | 57.18 ± 12.0 | 62.16 ± 8.26 | 53.49 ± 12.94 | 71.33 ± 3.73 | 71.20 ± 3.87 | 71.00 ± 3.89 |
| Smoking status | NA | | NA | - |  | Disc. | Disc. |  |  |  |  | Disc. |  |  |  |  |  |  |  |  |  |  | NA | NA | NA |
| Current | - | | - | - | 37  (64.9) | - | - | 659 (56.6) | 663 (48.3) | 333 (43.8) | 46  (70.8) | - | 5  (55.6) | 91 (17.9) | 77 (18.4) | 106 (17.8) | 0  (0) | 0  (0) | 0  (0) | 60 (14.0) | 64 (12.7) | 148 (14.4) | - | - | - |
| Former | - | | - | - | 6  (10.5) | - | - | 368 (31.6) | 573 (41.7) | 369 (48.5) | 14  (21.5) | - | 4  (44.4) | 194 (38.3) | 198 (47.4) | 285 (47.9) | 0  (0) | 0  (0) | 0  (0) | 169 (39.3) | 252 (50.0) | 446 (43.5) | - | - | - |
| Never | - | | - | - | 9  (15.8) | - | - | 125 (10.7) | 123  (9.0) | 52 (6.8) | 5  (7.7) | - | 0  (0) | 208 (41.0) | 134 (32.1) | 179 (30.1) | 50  (100) | 26  (100) | 9  (100) | 199 (46.3) | 186 (36.3) | 422 (41.2) | - | - | - |
| Education, >12 y | NA | | NA | - | 26  (45.6) | 10 (71.4) | Disc. | 566 (48.6) | 632 (46) | 297 (39) | NA | NA | NA | 291 (57.4) | 174 (41.2) | 159 (26.7) | NA | NA | NA | 223 (51.9) | 195 (38.7) | 390 (38.0) | NA | NA | NA |
| Prevalent cardiometabolic diseases^b^ | 0  (0) | | 0  (0) | - | NA | NA | NA | 1141 (98) | 1365 (99.3) | Disc. | 6  (9.2) | 6  (18.2) | 6  (66.7) | 108 (21.3) | 174 (41.6) | 385 (64.7) | 27  (54) | 20 (76.92) | Disc. | 367 (85.3) | 493 (97.8) | 981 (95.7) | NA | NA | NA |
| Current use of medication^c^ | 0  (0) | | 0  (0) | - | NA | NA | NA | 324 (27.8) | 609 (44.3) | 421 (55.3) | 5  (7.7) | Disc. | Disc. | 54 (10.7) | 69 (16.5) | 208 (35) | 0  (0) | Disc. | Disc. | 134 (31.2) | 355 (70.4) | 522 (50.9) | NA | NA | NA |
| Regular use of probiotics | 0  (0) | | 0  (0) | - | NA | NA | NA | NA | NA | NA | NA | NA | NA | NA | NA | NA | 0  (0) | 0  (0) | 0  (0) | NA | NA | NA | NA | NA | NA |
| Recent use of antibiotics^d^ | 125 (100) | | 18  (100) | - | 16  (28.1) | Disc. | Disc. | 0  (0) | 6  (0.4) | 4  (0.5) | 4  (6.2) | 4  (12.1) | 0  (0) | NA | NA | NA | 50  (100) | 26  (100) | 9  (100) | 169 (39.3) | 174 (34.5) | 477 (46.5) | NA | NA | NA |
| Physical activity, MET-h/day^e^ | NA | | NA | - | 5.45 [4.01, 7.32] | 6.18 [4.11, 9.43] | 6.37 [3.69, 8.54] | NA | NA | NA | NA | NA | NA | 1.96 [1.15, 3.36] | 2.29 [1.25, 3.56] | 2.36 [1.36, 4.04] | NA | NA | NA | 11.51 [7.22, 18.0] | 11.62 [6.78, 19.83] | 9.86 [5.27, 17.32] | NA | NA | NA |
| Dietary intake |  | |  |  |  |  |  |  |  |  |  |  |  |  |  |  |  |  |  |  |  |  |  |  |  |
| Total energy, kcal/d | 2324 [2097, 2540] | | 2732 [2380, 2962] | - | 1548 [1404, 1674] | 1582 [1375, 1770] | 1552 [1465, 1818] | NA | NA | NA | 1765 [1479, 2622] | 2075 [1545, 2963] | 1785 [1454, 2539] | 2078 [1753, 2493] | 2054 [1696, 2548] | 2061 [1669, 2606] | 1658 [1598, 1797] | 1700 [1610, 1873] | 1817 [1664, 2018] | NA | NA | NA | 1742 [1522, 1993] | 1760 [1502, 2042] | 1652 [1336, 1952] |
| Carbohydrate intake, g/d | 304.02 [265.88, 352.45] | | 337.80 [283.66, 384.74] | - | 193.93 [171.30, 211.74] | 195.71 [171.46, 231.23] | 197.15 [182.98, 243.13] | NA | NA | NA | 248.84 [173.69, 348.68] | 263.90 [178.11, 389.81] | 236.21 [159.31, 336.27] | 215.62 [176.81, 268.18] | 203 [168.92, 264.84] | 220.24 [167.44, 280.39] | 204.91 [179.21, 229.19] | 205.92 [179.23, 235.77] | 218.35 [194.40, 232.27] | NA | NA | NA | 229.06 [183.88, 271.60] | 228.15 [178.04, 258.39] | 200.78 [150.64, 253.63] |
| Fibre intake, g/d | 37.04 [26.61, 46.78] | | 41.15 [28.51, 53.21] | - | 14.27 [12.94, 15.79] | 14.75 [12.70, 17.67] | 16.23 [15.61, 19.54] | NA | NA | NA | 22.75 [17.34, 35.04] | 26.69 [15.60, 36.63] | 20.20 [14.23, 25.06] | 21.23 [17.33, 27.03] | 20.34 [16.57, 25.15] | 20.65 [16.49, 25.86] | 15.73 [13.45, 21.72] | 16.18 [13.42, 20.31] | 16.77 [14.68, 21.44] | NA | NA | NA | 23.11 [16.90, 28.33] | 20.41 [15.29, 26.27] | 19.76 [14.00, 24.18] |
| Protein intake, g/d | 73.25 [64.02, 84.83] | | 95.27 [80.24, 109.38] | - | 49.28 [42.97, 54.29] | 49.81 [45.75, 58.61] | 57.78 [55.51, 64.52] | NA | NA | NA | 71.42 [54.56, 95.77] | 83.69 [62.49, 108.2] | 66.39 [49.92, 97.62] | 72.31 [59.57, 89.52] | 75.48 [61.5, 94.72] | 77.94 [62.69, 98.79] | 67.5 [60.24, 78.18] | 68.51 [61.71, 80.58] | 72.39 [64.24, 77.33] | NA | NA | NA | 66.96 [59.01, 72.78] | 68.14 [57.19, 77.70] | 67.69 [56.93, 78.31] |
| Total fat intake, g/d | 93.35 [81.58, 105.62] | | 101.02 [91.62, 131.01] | - | 58.80 [55.29, 66.54] | 59.53 [52.56, 69.85] | 58.60 [56.74, 62.62] | NA | NA | NA | 64.62 [44.64, 82.86] | 75.81 [56.56, 93.67] | 57.17 [51.87, 84.94] | 92.06 [75.54, 116.35] | 94.58 [76.53, 115.69] | 90.66 [73.01, 110.48] | 71.73 [65.69, 77.65] | 71.95 [66.56, 77.35] | 68.69 [66.57, 73.37] | NA | NA | NA | 58.32 [52.36, 75.16] | 60.23 [51.61, 70.87] | 58.00 [49.72, 67.17] |
| Saturated fatty acid intake, g/d | 23.75 [16.42, 35.28] | | 30.75 [17.76, 43.97] | - | 27.75 [24.08, 30.77] | 26.85 [23.66, 31.24] | 25.51 [24.64, 30.19] | NA | NA | NA | 29.09 [21.34, 36.91] | 33.65 [24.16, 42.38] | 28.48 [23.44, 37.45] | 37.76 [30.14, 48.1] | 38.17 [30.49, 46.85] | 36.18 [28.61, 45.25] | 17.63 [13.77, 19.85] | 20.07 [16.69, 21.59] | 18.59 [15.27, 22.9] | NA | NA | NA | 18.14 [14.71, 21.36] | 19.04 [15.81, 23.87] | 18.64 [15.32, 23.92] |
| Monounsaturated fatty acid intake, g/d | 51.04 [44.34, 57.47] | | 56.40 [50.22, 65.64] | - | 19.70 [17.91, 22.53] | 20.31 [17.10, 23.37] | 20.45 [18.00, 21.64] | NA | NA | NA | 20.36 [15.02, 28.00] | 26.08 [20.22, 29.51] | 18.95 [16.54, 29.00] | 15.17 [12.4, 19.56] | 16.08 [12.91, 20.04] | 15.78 [12.74, 19.07] | 34.13 [29.6, 37.6] | 31.22 [28.89, 34.69] | 27.92 [25.05, 29.66] | NA | NA | NA | 27.50 [21.90, 32.71] | 27.37 [22.28, 32.42] | 26.68 [23.00, 30.75] |
| Polyunsaturated fatty acid intake, g/d | 15.37 [12.68, 19.53] | | 16.94 [14.69, 21.20] | - | 7.91 [6.95, 9.00] | 7.89 [6.98, 9.45] | 8.50 [7.82, 9.23] | NA | NA | NA | 8.42 [6.01, 12.41] | 9.53 [8.05, 14.38] | 7.21 [6.40, 9.53] | 31.99 [25.98, 40.79] | 33.59 [27.21, 41.5] | 31.78 [25.48, 39.33] | 7.92 [6.98, 9.03] | 8.25 [6.78, 10.57] | 6.92 [5.66, 11.4] | NA | NA | NA | 8.26 [6.70, 10.99] | 7.70 [6.27, 10.16] | 7.32 [5.92, 9.07] |
| Nondrinkers | 27 (22.6) | | disc.(<3 drank) | - | 0  (0) | 0  (0) | 0  (0) | NA | NA | NA | 0  (0) | 0  (0) | 0  (0) | 0  (0) | 0  (0) | 0  (0) | NA | NA | NA | NA | NA | NA | 11  (18) | 20 (19.6) | 11 (29.7) |
| Alcohol, g/d | 4.68 [0.25, 9.91] | | 4.17 [2.42, 16.88] | - | 0.49 [0.31, 1.38] | 1.37 [0.50, 2.43] | 0.61 [0.45, 1.72] | NA | NA | NA | 5.12 [2.20, 9.50] | 6.23 [1.17, 10.39] | 4.13 [3.27, 8.26] | 8.14 [3.23, 17.07] | 8.09 [3.01, 17.47] | 2.67 [1.14, 7.91] | NA | NA | NA | NA | NA | NA | 5.87 [0.83, 15.11] | 5.30 [0.78, 15.32] | 4.35 [0.00, 10.26] |
| Gut microbiota composition | | | |  |  |  |  |  |  |  |  |  |  |  |  |  |  |  |  |  |  |  |  |  |  |
| Shannon-Weiner alpha diversity index | 5.96 [5.38, 6.44] | | 5.52 [4.80, 6.19] | - | 6.18 [5.87, 6.38] | 6.10 [5.60, 6.27] | 5.83 [5.58, 5.97] | 4.27 [4.02, 4.49] | 4.26 [4.02, 4.49] | 4.18 [3.90, 4.43] | 4.27 [4.07, 4.47] | 4.18 [3.99, 4.47] | 4.18 [3.72, 4.47] | 4.23 [3.88, 4.53] | 4.23 [3.88, 4.53] | 4.00 [3.65, 4.37] | 4.17 [3.95, 4.39] | 4.28 [3.97, 4.55] | 4.30 [4.21, 4.45] | 3.78 [3.43, 3.98] | 3.58 [3.15, 3.88] | 3.40 [2.97, 3.76] | 2.01 [1.64, 2.17] | 2.02 [1.68, 2.20] | 1.78 [1.54, 2.05] |
| *F:B* ratio | 1.61 [0.92, 2.99] | | 1.60 [0.77, 2.62] | - | 1.91 [1.45, 2.19] | 1.87 [1.63, 2.42] | 1.42 [1.37, 1.57] | 1.49 [1.16, 1.95] | 1.38 [1.10, 1.82] | 1.26 [0.98, 1.69] | 4.18 [2.76, 6.59] | 4.63 [2.17, 6.38] | 9.02 [3.25, 21.50] | 1.04 [0.75, 1.44] | 0.95 [0.71, 1.31] | 1.06 [0.70, 1.78] | 1.80 [1.24, 2.50] | 1.94 [1.19, 2.72] | 2.86 [1.32, 5.32] | 0.57 [0.34, 0.92] | 0.52 [0.29, 0.85] | 0.46 [0.26, 0.81] | 13.70 [4.50, 53.85] | 16.60 [7.00, 54.42] | 20.87 [10.04, 305.65] |
| *P:B* ratio, high (>0.01) | 65 (52.0) | | 10  (55.6) | - | 0  (0) | 0  (0) | 0  (0) | 30  (2.6) | 42  (3.1) | 22  (2.9) | 28  (43.1) | 15  (45.5) | 5  (55.6) | 185 (36.5) | 181 (43.3) | 287 (48.2) | 3 (6) | Disc. | Disc. | 173 (40.2) | 234 (46.4) | 423 (41.3) | 36  (59.0) | 55  (53.9) | 16  (43.2) |

*F:B* ratio, Firmicutes to *Bacteroidetes* ratio; *P:B* ratio, *Prevotella* to *Bacteroides* ratio; disc., disclosive.

^a^ Values are mean ± SD or median [25^th^, 75^th^ percentiles] or counts (%).

^b^ Hypertension, diabetes, cardiovascular diseases, and dyslipidemia. No data on dyslipidemia available for FoCus.

^c^ Statins, non-steroidal anti-inflammatory drugs (NSAIDs), and/or diabetes medications. No data on NSAID available for MeaTIc and MetaCardis.

^d^ Study-specific definitions: Diet4MicroGut and ErNst, past 12+ months; DONALD and MeaTIc, past 6 months; EPIC-Potsdam substudy, past 3 months; MetaCardis, past 5 years up to 3 months before enrollment.

^e^ DONALD and FoCus: total physical activity (excludes sedentary and sleeping time); MetaCardis: leisure-time physical activity.

SUPPPLEMENTAL TABLE 4: Taxa prevalence and distribution in samples across eight European studies (N=7,415)

| **Taxa** | **All studies (n=8)** | **Diet4MicroGut** | | **DONALD** | | **EPIC-Potsdam substudy** | | **ErNst** | | **FoCus** | | **MeaTIc** | | **MetaCardis** | | **NU-AGE** | |
| --- | --- | --- | --- | --- | --- | --- | --- | --- | --- | --- | --- | --- | --- | --- | --- | --- | --- |
|  | **Prevalence**  **N (%)** | **Prevalence**  **N (%)** | **Median [IQR]** | **Prevalence**  **N (%)** | **Median [IQR]** | **Prevalence**  **N (%)** | **Median [IQR]** | **Prevalence**  **N (%)** | **Median [IQR]** | **Prevalence**  **N (%)** | **Median [IQR]** | **Prevalence**  **N (%)** | **Median [IQR]** | **Prevalence**  **N (%)** | **Median [IQR]** | **Prevalence**  **N (%)** | **Median [IQR]** |
| **PHYLUM** | | | | | | | | | | | | | | | | | |
| *Actinobacteria* | 7318 (99) | 142 (99) | 0.81  [0.30, 2.41] | 79 (100) | 4.19  [2.85, 6.12] | 3287 (100) | 1.48  [0.68, 3.07] | 107 (100) | 3.12  [1.79, 5.86] | 1471 (97) | 0.23  [0.07, 0.65] | 85 (100) | 3.22  [1.80, 8.24] | 1977 (100) | 0.48  [0.21, 0.98] | 170 (85) | 4.61  [0.76, 10.42] |
| *Bacteroidetes* | 2564 (99)^a^ | 142 (99) | 34.49  [22.90, 49.84] | 79 (100) | 32.26  [27.4, 38.08] | discl.(<3 zero) | 36.66  [30.90, 42.68] | 107 (100) | 17.22  [11.26, 25.34] | discl.(<3 zero) | 42.9  [34.21, 51.71] | 85 (100) | 32.49  [24.43, 41.29] | 1982 (100) | 31.79  [20.44, 47.93] | 169 (84.5) | 5.1  [1.25, 11.56] |
| *Firmicutes* | 5894 (100) ^a^ | 142 (99) | 56.42  [43.79, 69.42] | 79 (100) | 57.26  [52.77, 62.60] | 3299 (100) | 51.58  [45.63, 57.10] | 107 (100) | 73.83  [66.17, 80.58] | discl.(<3 zero) | 43.69  [35.14, 53.25] | 85 (100) | 58.99  [51.54, 68.79] | 1982 (100) | 15.14  [11.17, 19.84] | 200 (100) | 84.08  [74.87, 90.97] |
| *Proteobacteria* | 3801 (97) ^a^ | 137 (96) | 0.30  [0.11, 0.72] | 79 (100) | 2.89  [1.83, 4.00] | 3296 (100) | 4.74  [2.88 , 8.03] | 107 (100) | 0.58  [0.33, 1.13] | discl.(<3 zero) | 8.11  [3.57, 16.16] | 85 (100) | 0.64  [0.24, 1.12] | discl.(<3 zero) | 0.95  [0.44, 1.99] | 97 (48.5) | 0.00  [0.00, 1.07] |
| *Verrucomicrobia* | 4806 (66) ^a^ | 23 (16) | 0.00  [0.00, 0.00] | discl.(<3 zero) | 0.07  [0.00, 0.84] | 2577 (78) | 0.40  [0.03, 1.74] | 91 (85) | 0.27  [0.02, 2.21] | 878 (58) | 0.02  [0.00, 0.23] | 76 (89.41) | 0.08  [0.003, 1.87] | 1122 (57) | 0.01  [0.00, 0.69] | 39 (19.5) | 0.00  [0.00, 0.00] |
| **GENUS** | | | | | | | | | | | | | | | | | |
| *Bifidobacterium* | 5651 (76) | 10 (7) | 0.00  [0.00, 0.00] | 79 (100) | 2.64  [1.45, 4.06] | 2894 (88) | 0.62  [0.15, 1.78] | 104 (97) | 1.94  [0.78, 3.60] | 571 (38) | 0.00  [0.00, 0.02] | 85 (100) | 1.63  [0.80, 4.67] | 1802 (91) | 0.26  [0.06, 0.68] | 106 (53) | 0.72  [0.00, 7.71] |
| *Collinsella* | 6185 (83) | 121 (85) | 0.35  [0.08, 1.23] | 79 (100) | 0.79  [0.23, 1.63] | 2882 (87) | 0.31  [0.11, 0.79] | 92 (86) | 0.51  [0.07, 1.16] | 1164 (77) | 0.08  [0.01, 0.28] | 79 (92.94) | 0.53  [0.17, 1.38] | 1708 (86) | 0.14  [0.06, 0.28] | 60 (30) | 0.00  [0.00, 1.43] |
| *Bacteroides* | 5340 (98) ^a^ | 143 (100) | 14.22  [3.50, 25.24] | 79 (100) | 19.66  [14.23, 25.68] | 3291 (100) | 17.17  [9.52, 25.30] | 107 (100) | 8.19  [4.02, 13.57] | 1516 (100) | 29.27  [18.15, 40.82] | 85 (100) | 16.87  [11.24, 24.73] | discl.(<3 zero) | 13.42  [6.25, 25.99] | 119 (59.5) | 1.43  [0.00, 5.03] |
| *Odoribacter* | 6374 (87) ^a^ | 88 (62) | 0.03  [0.00, 0.09] | discl.(<3 zero) | 0.45  [0.30, 0.68] | 3039 (92) | 0.29  [0.15, 0.48] | 98 (92) | 0.07  [0.03, 0.12] | 1242 (82) | 0.20  [0.04, 0.44] | 81 (95.29) | 0.12  [0.04, 0.22] | 1823 (92) | 0.36  [0.18, 0.55] | 3 (1.5) | 0.00  [0.00, 0.00] |
| *Parabacteroides* | 6683 (93) ^a^ | 138 (97) | 1.15  [0.47, 2.06] | 79 (100) | 1.09  [0.65, 1.82] | 3127 (95) | 1.49  [0.82, 2.45] | discl.(<3 zero) | 0.59  [0.27, 1.03] | 1408 (93) | 2.23  [0.93, 3.97] | discl.(<3 zero) | 1.21  [0.63, 2.11] | 1889 (95) | 1.36  [0.68, 2.54] | 42 (21) | 0.00  [0.00, 0.00] |
| *Paraprevotella* | 1703 (23) | NA | NA | 47 (59) | 0.00  [0.00, 0.01] | 807 (24) | 0.00  [0.00, 0.00] | 58 (54) | 0.001  [0.00, 0.28] | 572 (38) | 0.00  [0.00, 0.56] | 59 (69.41) | 0.09  [0.00, 0.62] | 160 (8) | 0.00  [0.00, 0.00] | 0 (0) | 0.00  [0.00, 0.00] |
| *Prevotella* | 2725 (37) | 84 (59) | 0.14  [0.00, 19.11] | 50 (63) | 0.00  [0.00, 0.00] | 270 (8) | 0.00  [0.00, 0.00] | 85 (79) | 0.02  [0.001, 1.77] | 841 (55) | 0.02  [0.00, 6.47] | 26 (30.59) | 0.00  [0.00, 0.002] | 1336 (67) | 0.01  [0.00, 9.49] | 33 (16.5) | 0.00  [0.00, 0.00] |
| *Rikenella* | 255 (5) | NA | NA | 39 (49) | 0.00  [0.00, 0.00] | 90 (3) | 0.00  [0.00, 0.00] | 8 (7) | 0.00  [0.00, 0.00] | 91 (6) | 0.00  [0.00, 0.00] | 12 (14.12) | 0.00  [0.00, 0.00] | NA | NA | 15 (7.5) | 0.00  [0.00, 0.00] |
| *Blautia* | 5492 (74) | 143 (100) | 5.13  [2.59, 9.90] | 79 (100) | 5.01  [3.88, 6.92] | 3284 (100) | 0.64  [0.39, 1.04] | 107 (100) | 8.59  [6.11, 13.43] | 1434 (94) | 0.30  [0.13, 0.71] | 85 (100) | 2.37  [1.20,4. 29] | 285 (14) | 0.00  [0.00, 0.00] | 75 (37.5) | 0.00  [0.00, 8.68] |
| *Clostridium* | 3686 (51) ^a^ | discl.(<3 zero) | 0.47  [0.30, 0.84] | NA | NA | 10 (0) | 0.00  [0.00, 0.00] | 101 (94) | 0.40  [0.08, 1.01] | 1513 (100) | 2.08  [1.12, 3.73] | 79 (92.94) | 0.06  [0.02, 0.19] | 1943 (98) | 0.11  [0.03, 0.41] | 40 (20) | 0.00  [0.00, 0.00] |
| *Coprococcus* | 6377 (87) ^a^ | 143 (100) | 3.37  [1.75, 5.53] | 79 (100) | 0.16  [0.09, 0.22] | 3071 (93) | 0.91  [0.37, 1.75] | 107 (100) | 1.83  [0.64, 3.19] | 1175 (77) | 0.14  [0.02, 0.44] | discl.(<3 zero) | 1.04  [0.30, 1.75] | 1802 (91) | 0.24  [0.12, 0.43] | 0 (0) | 0.00  [0.00, 0.00] |
| *Dialister* | 4210 (57) ^a^ | 102 (71) | 0.31  [0.00, 1.71] | discl.(<3 zero) | 0.76  [0.01, 1.88] | 2048 (62) | 0.98  [0.00, 3.47] | 88 (82) | 1.01  [0.003, 2.94] | 1133 (75) | 3.59  [0, 11.72] | 72 (84.71) | 0.24  [0.002, 1.89] | 726 (37) | 0.00  [0.00, 0.34] | 41 (20.5) | 0.00  [0.00, 0.00] |
| *Dorea* | 6397 (89) ^a^ | 17 (12) | 0.00  [0.00, 0.00] | discl.(<3 zero) | 0.14  [0.08, 0.22] | 3096 (94) | 0.26  [0.13, 0.50] | discl.(<3 zero) | 1.06  [0.66, 1.66] | 1266 (83) | 0.12  [0.04, 0.31] | 85 (100) | 1.08  [0.49, 2.23] | 1890 (95) | 0.20  [0.11, 0.33] | 43 (21.5) | 0.00  [0.00, 0.00] |
| *Eubacterium* | 2290 (31) | 117 (82) | 0.07  [0.02, 0.15] | 12 (15) | 0.00  [0.00, 0.00] | 24 (0.7) | 0.00  [0.00, 0.00] | 6 (6) | 0.00  [0.00, 0.00] | 33 (2) | 0.00  [0.00, 0.00] | 6 (7.06) | 0.00  [0.00, 0.00] | 1951 (98) | 2.66  [1.25, 4.83] | 141 (70.5) | 13.15  [0.00, 28.94] |
| *Faecalibacterium* | 7124 (96) | 143 (100) | 10.89  [6.66, 13.66] | 79 (100) | 8.58  [6.13, 12.33] | 3234 (98) | 6.32  [4.03, 9.01] | 107 (100) | 3.23  [1.78, 5.26] | 1451 (95) | 3.80  [1.38, 8.56] | 85 (100) | 7.55  [4.2, 11.73] | 1912 (96) | 3.56  [2.07, 5.46] | 113 (56.5) | 4.65  [0.00, 13.08] |
| *Lachnospira* | 3458 (66) ^a^ | 138 (97) | 0.43  [0.18, 1.14] | 79 (100) | 0.85  [0.50, 1.55] | 3193 (97) | 1.37  [0.63, 2.35] | discl.(<3 zero) | 1.10  [0.52, 2.43] | 24 (2) | 0.00  [0.00, 0.00] | discl.(<3 zero) | 0.17  [0.05, 0.54] | NA | NA | 24 (12) | 0.00  [0.00, 0.00] |
| *Oscillospira* | 1678 (43) | 133 (93) | 0.42  [0.12, 0.83] | 71 (90) | 0.02  [0.01, 0.04] | 1369 (42) | 0.00  [0.00, 0.07] | 85 (79) | 0.01  [0.002, 0.04] | NA | NA | 20 (23.53) | 0.00  [0.00, 0.00] | NA | NA | 0 (0) | 0.00  [0.00, 0.00] |
| *Roseburia* | 6969 (94) | 143 (100) | 2.10  [1.18, 3.84] | 79 (100) | 1.18  [0.80, 2.23] | 3157 (96) | 1.20  [0.52, 2.33] | 107 (100) | 2.10  [0.92, 3.82] | 1454 (96) | 0.90  [0.34, 1.94] | 85 (100) | 3.79  [1.64, 6.84] | 1908 (96) | 0.77  [0.32, 1.61] | 36 (18) | 0.00  [0.00, 0.00] |
| *Ruminococcus* | 6956 (94) | 143 (100) | 0.69  [0.38, 1.16] | 79 (100) | 2.07  [1.13, 2.84] | 3146 (95) | 2.53  [1.12, 4.88] | 107 (100) | 4.23  [2.28, 6.56] | 1295 (85) | 0.53  [0.12, 1.46] | 85 (100) | 3.97  [1.76, 7.83] | 1976 (100) | 1.22  [0.54, 2.31] | 125 (62.5) | 2.95  [0.00, 8.61] |
| *Streptococcus* | 6227 (84) | 117 (82) | 0.14  [0.03, 0.55] | 79 (100) | 0.37  [0.16, 0.73] | 2533 (77) | 0.07  [0.02, 0.23] | 107 (100) | 0.30  [0.14, 0.69] | 1302 (86) | 0.12  [0.03, 0.44] | 85 (100) | 0.19  [0.08, 0.48] | 1945 (98) | 0.06  [0.02, 0.17] | 59 (29.5) | 0.00  [0.00, 0.54] |
| *Subdoligranulum* | 5102 (71) ^a^ | NA | NA | 79 (100) | 2.23  [1.17, 3.17] | 3152 (96) | 1.88  [0.92, 3.40] | discl.(<3 zero) | 1.32  [0.66, 2.40] | 1413 (93) | 2.04  [0.75, 4.15] | 85 (100) | 3.37  [2.03, 5.59] | 250 (13) | 0.00  [0.00, 0.00] | 123 (61.5) | 9.12  [0.00, 19.59] |
| *Veillonella* | 3446 (48) ^a^ | 39 (27) | 0.00  [0.00, 0.02] | discl.(<3 zero) | 0.03  [0.01, 0.05] | 1244 (38) | 0.00  [0.00, 0.05] | 96 (90) | 0.03  [0.01, 0.11] | 627 (41) | 0.00  [0.00, 0.05] | NA | NA | 1426 (72) | 0.01  [0.00, 0.05] | 14 (7) | 0.00  [0.00, 0.00] |
| *Enterobacter* | 712 (10) ^a^ | NA | NA | 57 (72) | 0.00  [0.00, 0.02] | 234 (7) | 0.00  [0.00, 0.00] | 0 (0) | 0.00  [0.00, 0.00] | 88 (6) | 0.00  [0.00, 0.00] | NA | NA | 333 (17) | 0.00  [0.00, 0.00] | discl.(<3 nonzero) | 0.00  [0.00, 0.00] |
| *Sutterella* | 4326 (58) | 63 (44) | 0.00  [0.00, 0.06] | 76 (96) | 0.85  [0.01, 1.41] | 2391 (72) | 0.85  [0.00, 2.06] | 84 (79) | 0.07  [0.003, 0.23] | 809 (53) | 0.02  [0.00, 1.00] | 40 (47.06) | 0.00  [0.00, 0.01] | 848 (43) | 0.00  [0.00, 0.35] | 15 (7.5) | 0.00  [0.00, 0.00] |
| *Akkermansia* | 4680 (63) | 23 (16) | 0.00  [0.00, 0.00] | 76 (96) | 0.06  [0.00, 0.73] | 2436 (74) | 0.31  [0.00, 1.61] | 90 (84) | 0.24  [0.01, 2.21] | 874 (58) | 0.02  [0.00, 0.32] | 20 (23.53) | 0.00  [0.00, 0.00] | 1122 (57) | 0.01  [0.00, 0.69] | 39 (19.5) | 0.00  [0.00, 0.00] |

^a^ Prevalence shown excludes studies with disclosive results (<3 observations in a category).

SUPPLEMENTAL TABLE 5: Individual person data analyses of the associations between BMI (per 5-unit increase) and alpha diversity, relative abundance of prevalent taxa on the phylum and genus level^a^ and F:B and P:B ratios

| Gut microbiome composition characteristic | Mean difference per 5 BMI (kg/m^2^)-units increase^b^ | | | |
| --- | --- | --- | --- | --- |
|  | N | β (95% CI) | Adjusted P value^c^ | |
| Alpha diversity | 7175 | -0.06 (-0.07; -0.05) | <0.0001 | |
| F:B ratio^d^ | 7385 | -0.02 (-0.03; -0.01) | 0.004 | |
|  |  |  |  | |
| Prevalent taxa (present in >90% of samples) | | | | |
| Phlyum |  |  |  |  |
| Actinobacteria^d^ | 7385 | 0.04 (0.02; 0.06) | 0.001 | |
| Bacteroidetes | 7385 | 0.69 (0.44; 0.94) | <0.0001 | |
| Firmicutes | 7385 | -0.07 (-0.26; 0.11) | 0.57 | |
| Proteobacteria^d^ | 7385 | 0.05 (0.03; 0.07) | <0.0001 | |
| Genus |  |  |  | |
| *Bacteroides* (*Bacteroidetes*)^d^ | 7386 | 0.00 (-0.02; 0.02) | 0.91 | |
| *Parabacteroides* (*Bacteroidetes*)^d^ | 7386 | -0.02 (-0.05; 0.01) | 0.18 | |
| *Faecalibacterium* (*Firmicutes*)^d^ | 7386 | -0.12 (-0.14; -0.09) | <0.0001 | |
| *Roseburia* (*Firmicutes*)^d^ | 7386 | 0.01 (-0.01; 0.04) | 0.36 | |
| *Ruminococcus* (*Firmicutes*)^d^ | 7386 | -0.07 (-0.10, -0.05) | <0.0001 | |
|  |  |  |  | |
|  | Odds per 5 BMI (kg/m^2^)-units increase^g^ | | | |
|  | N | OR (95% CI) | Adjusted P value^c^ | |
| *P:B* ratio (high vs. low)^h^ | 7386 | 1.09 (1.05; 1.13) | <0.0001 | |
|  |  |  |  | |

F:B, Firmicutes to Bacteroidetes ratio; *P:B*, *Prevotella* to *Bacteroides* ratio.

^a^ Participants with alpha diversity of zero were excluded; N=4 in FoCus and N=1 in NU-AGE. Excluding taxa present in <10% of samples.

^b^ Linear regression using generalized linear models using robust standard errors to address heteroscedasticity of residuals observed in some models. All models are sex and age adjusted.

^c^ Using Benjamini-Hochberg FDR controlling procedure.

^d^ Ratios and taxa were log-transformed (natural logarithm). A pseudocount of 0.01% was added to relative abundance (all observations) to allow for log-transformation in the presence of zeros.


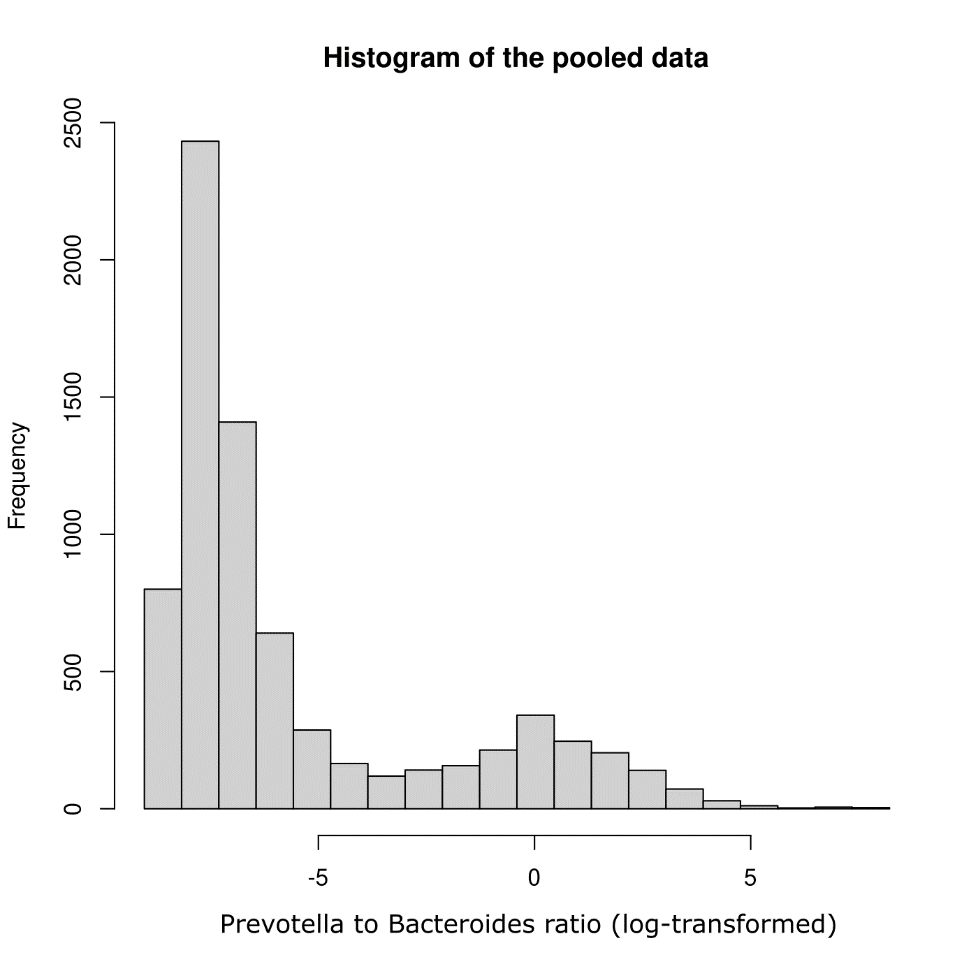


SUPPLEMENTAL FIGURE 1: Histogram of log-transformed *Prevotella* to *Bacteroides* ratio among adults from 8 European studies.


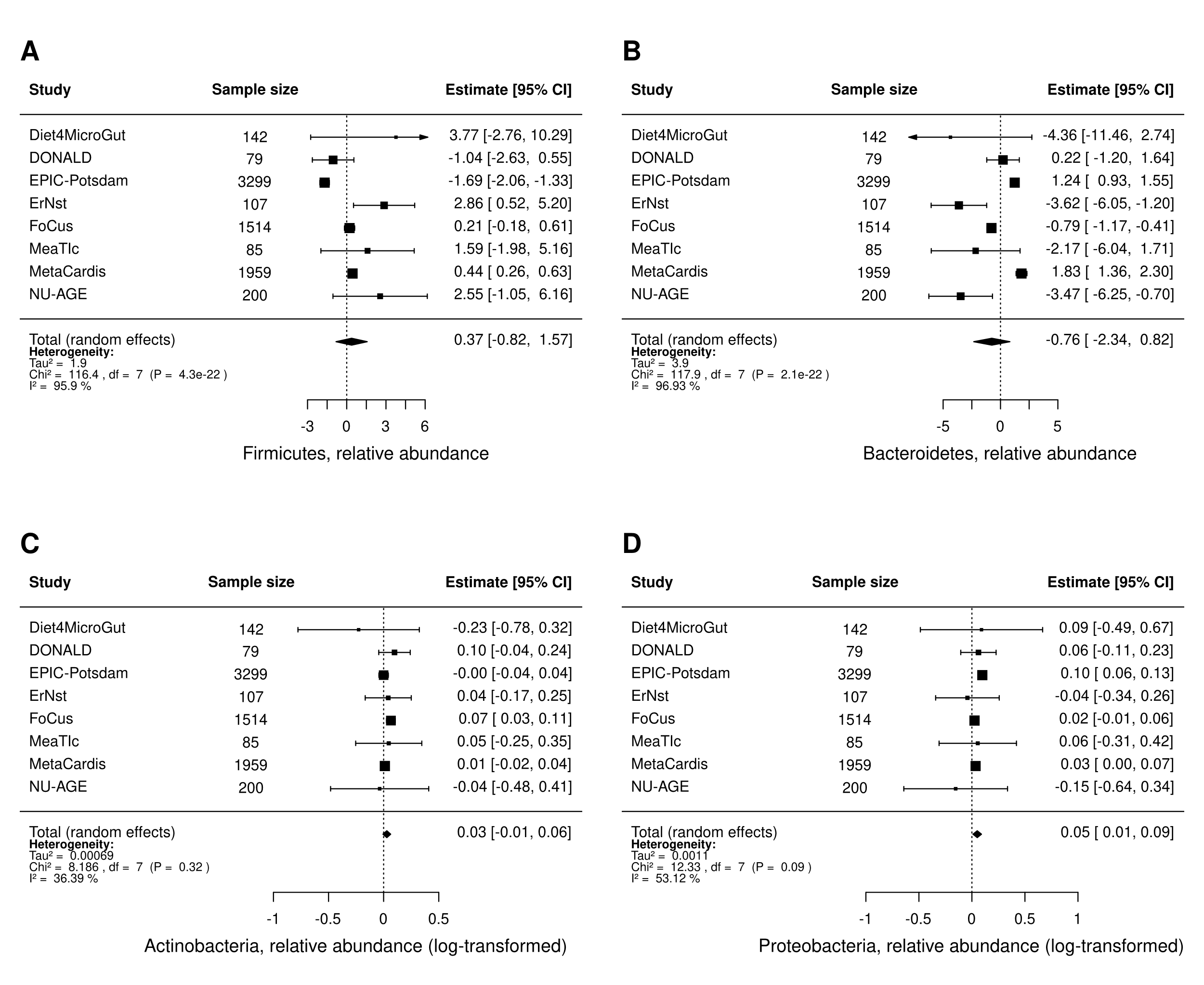


SUPPLEMENTAL FIGURE 2: Forest plots of random-effects study level meta-analysis among adults from 8 European studies, sex and age adjusted, showing the mean difference in relative abundance of prevalent taxa (present in >90% of samples) on the phylum level per 5-unit BMI increment. Phyla are A) Firmicutes, B) Bacteroidetes, C) Actinobacteria (log-transformed), and D) Proteobacteria (log-transformed).


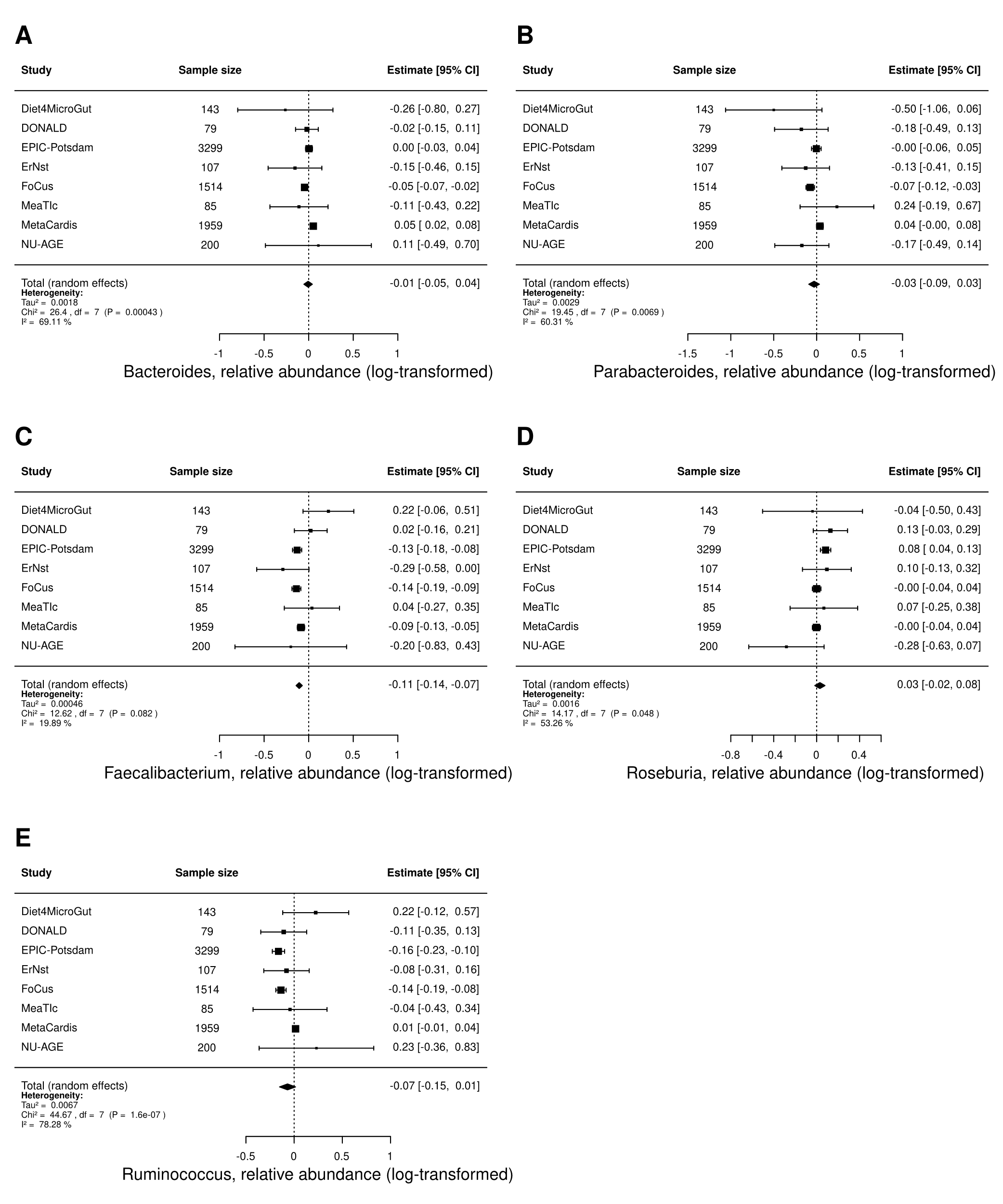


SUPPLEMENTAL FIGURE 3: Forest plots of random-effects study level meta-analysis among adults from 8 European studies, sex and age adjusted, showing the mean difference in log-transformed relative abundance of prevalent taxa (present in >90% of samples) on the genus level per 5-unit BMI increment. Genera are A) *Bacteroides*, B) *Proteobacteria*, C) *Faecalibacterium*, D) *Roseburia*, and E) *Ruminococcus*.
